# Transfer learning reveals sequence determinants of the quantitative response to transcription factor dosage

**DOI:** 10.1016/j.xgen.2025.100780

**Published:** 2025-02-27

**Authors:** Sahin Naqvi, Seungsoo Kim, Saman Tabatabaee, Anusri Pampari, Anshul Kundaje, Jonathan K. Pritchard, Joanna Wysocka

**Affiliations:** 1 Departments of Chemical and Systems Biology and Developmental Biology, Stanford University School of Medicine, Stanford, CA 94305, USA; 2 Department of Genetics, Stanford University, Stanford, CA 94305, USA; 3 Division of Gastroenterology, Hepatology, and Nutrition, Boston Children’s Hospital, Boston, MA 02115, USA; 4 Department of Pediatrics, Harvard Medical School, Boston, MA 02115, USA; 5 Howard Hughes Medical Institute, Stanford University School of Medicine, Stanford, CA 94305, USA; 6 Department of Computer Science, Stanford University, Stanford, CA 94305, USA; 7 Department of Biology, Stanford University, Stanford, CA 94305, USA

**Keywords:** gene regulation, transcription factors, gene dosage, degrons, deep learning, nucleosomes, chromatin accessibility, motif affinity

## Abstract

Deep learning models have advanced our ability to predict cell-type-specific chromatin patterns from transcription factor (TF) binding motifs, but their application to perturbed contexts remains limited. We applied transfer learning to predict how concentrations of the dosage-sensitive TFs TWIST1 and SOX9 affect regulatory element (RE) chromatin accessibility in facial progenitor cells, achieving near-experimental accuracy. High-affinity motifs that allow for heterotypic TF co-binding and are concentrated at the center of REs buffer against quantitative changes in TF dosage and predict unperturbed accessibility. Conversely, low-affinity or homotypic binding motifs distributed throughout REs drive sensitive responses with minimal impact on unperturbed accessibility. Both buffering and sensitizing features display purifying selection signatures. We validated these sequence features through reporter assays and demonstrated that TF-nucleosome competition can explain low-affinity motifs' sensitizing effects. This combination of transfer learning and quantitative chromatin response measurements provides a novel approach for uncovering additional layers of the *cis*-regulatory code.

## Highlights

• Transfer learning predicts chromatin response to transcription factor dosage
• Heterotypic motifs buffer and low-affinity motifs sensitize chromatin to dosage changes
• TF-specific models highlight shared and distinct sequence logic in chromatin response
• Biophysical modeling explains how nucleosome competition shapes dosage sensitivity

## Graphical abstract

**Figure:**
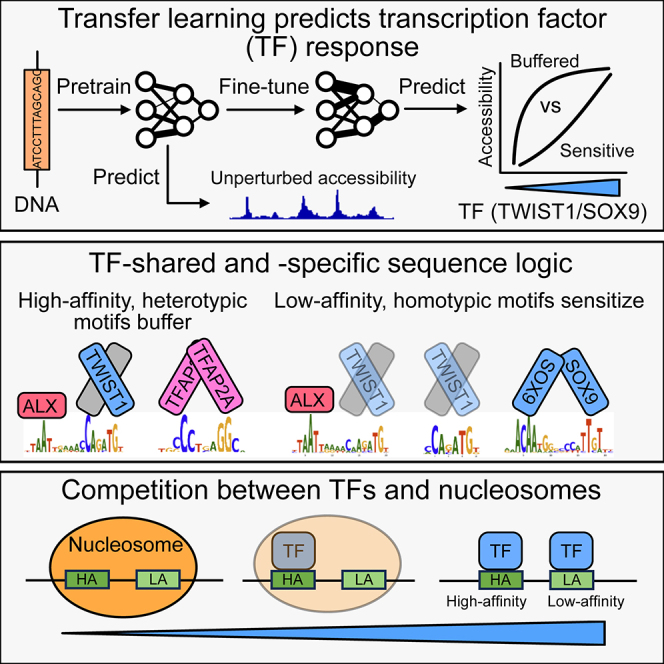

## Teaser

Naqvi et al. reveal how DNA sequence determines the chromatin response to transcription factor (TF) dosage changes. By combining deep learning and chemical genetics, they uncover specific sequence features that buffer or sensitize chromatin to TF dosage, offering insights into *cis*-regulatory logic and its implications for dosage-sensitive disorders.

## Introduction

Deciphering the *cis*-regulatory code, the rules by which DNA sequence encodes precise, timely, and context-specific gene expression, is a fundamental goal with broad utility in understanding, predicting, and ultimately treating human disease. One key aspect of the *cis*-regulatory code is chromatin state, the manner in which DNA is packaged in the nucleus. At short length scales (hundreds to thousands of base pairs), much of the cellular context-specific chromatin state is determined by transcription factors (TFs), proteins that bind to short DNA sequences termed motifs and, through either mass action or recruitment of enzymes that remodel and modify nucleosomes, set the activity of regulatory elements (REs) that subsequently modulate transcription of target genes.^1^^,^^2^ Thus, a key part of understanding the *cis*-regulatory code is the ability to predict chromatin state from the identity and arrangement of TF motifs.

Deep learning models have made substantial progress toward predicting RE chromatin states, as measured by genome-wide assays of histone modifications and chromatin accessibility, from DNA sequences.^3^^,^^4^^,^^5^^,^^6^^,^^7^ Neural network interpretation tools have revealed that much of this improvement in predictive power derives from flexibly encoding motif affinity and arrangement in a quantitative fashion.^6^^,^^7^ While such models implicitly learn the activity levels of relevant TFs in a given cell type, the vast majority of models to date have been trained to predict measurements of chromatin state in unperturbed cells, precluding a predictive understanding of how chromatin state responds to TF perturbation. Furthermore, TFs have been observed to be highly dosage sensitive in human variation and disease.^8^^,^^9^^,^^10^ Using deep learning to understand how chromatin state responds to quantitative changes in TF levels could, therefore, provide insights into transcriptional regulation while also furthering a mechanistic understanding of how variation in TF levels leads to both normal-range and disease-associated phenotypic diversity.

Predicting the chromatin response to TF dosage presents two main challenges. The first involves obtaining precise experimental measurements of the response to endogenous TF levels. We recently applied the degradation tag (dTAG) system to achieve precise modulation of SOX9 dosage in human facial progenitors (cranial neural crest cells [CNCCs]) derived *in vitro* from pluripotent stem cells.^11^ This revealed variable chromatin accessibility responses to SOX9 dosage, with most REs being buffered against quantitative SOX9 dosage changes but a subset showing highly sensitive responses. Sensitive responses were selectively linked to specific craniofacial phenotypes associated with SOX9 dosage perturbations, underscoring the importance of dosage effects for understanding human phenotypic variation. The basic-helix-loop-helix (bHLH) factor TWIST1 is another compelling candidate for the predictive analysis of dosage effects in CNCCs. Like SOX9, TWIST1 dosage changes are associated with normal-range and disease-related craniofacial variation in humans.^12^^,^^13^^,^^14^ We recently demonstrated that TWIST1 is a key driver of CNCC chromatin accessibility: depletion of TWIST1 in CNCCs results in reduced accessibility at >30,000 REs as a result of its binding to a composite motif termed “Coordinator” through cooperative, DNA-guided interactions with homeodomain TFs.^14^

The second challenge to predicting the chromatin response to TF dosage is the large number of training examples required by deep learning models. While steady-state models can use all detected REs (∼hundreds of thousands), only a subset of these REs are expected to respond to the perturbation of a single TF (≤tens of thousands), limiting the number of available training examples. Transfer learning, in which deep learning models are “pretrained” on a larger set of related examples and then fine-tuned to predict the desired task, has recently emerged as an attractive solution to this type of problem, enabling the use of deep learning in data-limited settings.^15^^,^^16^^,^^17^^,^^18^^,^^19^

Here, we combined the transfer learning of chromatin accessibility models with TF dosage titration by dTAG to learn the sequence logic underlying responsiveness to SOX9 and TWIST1 dosage in CNCCs. Our approach predicted how REs responded to TF dosage, both in magnitude and shape of response (sensitive or buffered), with accuracy approaching experimental reproducibility. Model interpretation revealed that composite or discrete motifs allowing for heterotypic TF interactions predict buffered responses, whereas low-affinity binding sites for TWIST1 predict sensitive responses. Despite their low importance in models of unperturbed cells, sensitizing sequences show similar conservation to buffering sequences. We experimentally validated this sequence logic and showed that TF-nucleosome competition explains the sensitizing effects of low-affinity sites.

## Results

### Precise modulation of TWIST1 dosage in hESC-derived CNCCs

**Figure 1:**
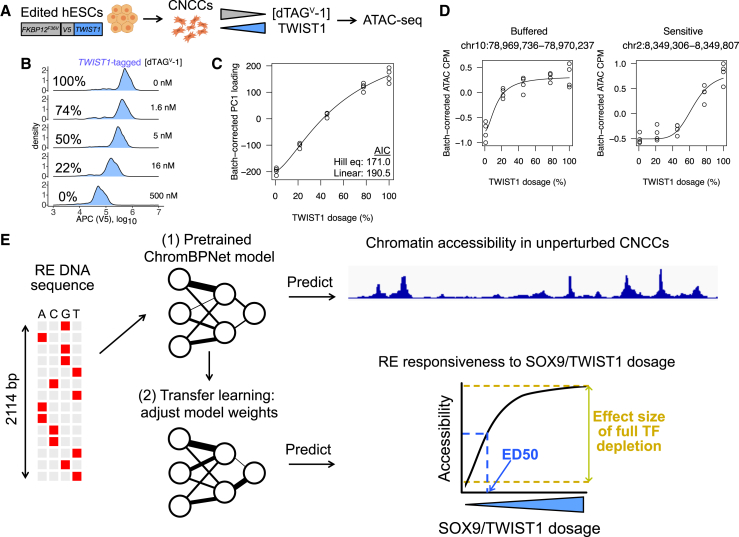
Approach to quantify and predict RE response to TF dosage (A) Schematic of approach for precise modulation of TWIST1 dosage. (B) Flow cytometry analysis of V5 staining intensity at 24 h in *TWIST1*-tagged CNCCs as a function of increasing dTAG^V^-1 concentrations, representative of two independent experiments (at least 5,000 cells per histogram). (C) Loadings from principal-component analysis (PCA) of ATAC-seq counts per million (CPM) of all 151,457 REs across all CNCC samples corrected for differentiation batch and plotted as a function of estimated relative TWIST1 dosage (shown as percentage relative to no dTAG^V^-1). (D) Examples of buffered and sensitive responses, with fitted Hill equation plotted. (E) Schematic of transfer learning approach to predict effect size of full depletion or ED_50_ for RE chromatin accessibility in response to SOX9 or TWIST1 dosage. See also Figure S1.

We first assessed whether the dTAG system, in which the FKBP12-F36V tag mediates target degradation following the addition of the dTAG^V^-1 small molecule, could be used to precisely modulate TWIST1 dosage in human embryonic stem cell (hESC)-derived CNCCs, as we previously did for SOX9.^11^ We used a previously generated hESC line with biallelic knockin of a FKBP12-F36V-V5 tag at the *TWIST1* N terminus.^14^ We differentiated *TWIST1*-tagged hESCs using an established protocol^20^^,^^21^ and subsequently titrated *TWIST1* levels by adding varying dTAG^V^-1 concentrations (Figure 1A). As *TWIST1* was not tagged with a fluorescent protein, we measured TWIST1 protein levels by intracellular staining with a monoclonal V5 antibody followed by flow cytometry. We confirmed linearity between intracellular V5 staining intensity and protein abundance by analyzing *SOX9*-tagged CNCCs, which also have the fluorescent mNeonGreen tag (Figure S1A). We achieved five distinct TWIST1 dosages after 24 h dTAG^V^-1 treatment, with unimodal single-cell distributions that shifted uniformly with increasing dTAG^V^-1 concentration (Figures 1B and S1B).

To assess the effect of TWIST1 dosage changes on chromatin accessibility, we carried out the assay for transposase-accessible chromatin with sequencing (ATAC-seq) on *TWIST1*-tagged CNCCs with five different TWIST1 dosages (four biological replicates at each dosage). We observed a nonlinear, monotonic effect of TWIST1 dosage in principal-component space (Figure 1C), and inspection of individual REs revealed that distinct responses to TWIST1 dosage ranged from buffered (minimal accessibility changes until TWIST1 dosage is greatly reduced) to sensitive (even small decreases in TWIST1 dosage from 100% leading to corresponding accessibility changes) (Figure 1D). Together, these results indicate that, as previously observed with SOX9, TWIST1 dosage effects on chromatin are largely monotonic but nonlinear and with sensitivity that can vary substantially between REs.

### Accurate prediction of RE responsiveness to TF dosage by transfer learning

We next sought to use transfer learning to predict RE responsiveness to TWIST1 and SOX9 dosage using two metrics to quantify the RE response. First, for all 151,457 ATAC peak regions, we calculated the log_2_ fold change in accessibility upon full TF depletion. Second, for each RE responding significantly to either SOX9 (35,712 REs as defined in Naqvi et al.^11^) or TWIST1 (50,850 REs at a 1% false discovery rate [FDR]) dosage, we calculated the median effective dose (ED_50_) of a fitted Hill equation. We slightly modified the ED_50_ calculation from Naqvi et al.^11^ due to the heightened sensitivity of some TWIST1-dependent REs leading to unstable estimates (see STAR Methods; Tables S1 and S2). Lower ED_50_ values indicate a more buffered response to decreases in TF dosage from 100%, whereas higher ED_50_ values indicate a more sensitive response (Figure 1E). Of all TWIST1-dependent REs, 20,222 (40%) have an ED_50_ < 30 (buffered), while 30,628 (60%) have an ED_50_ > 30 (sensitive). By the same definition, 27,805 (78%) of all SOX9-dependent REs are buffered, while 7,907 (22%) are sensitive. While TWIST1-dependent REs typically showed higher ED_50_ values than SOX9-dependent REs, there was still a substantial variation in both the full depletion effect and ED_50_ (Figure S2A). Furthermore, among REs dependent on both TWIST1 and SOX9 (18,416 REs; 32,442 and 17,297 REs dependent on only TWIST1 or SOX9, respectively), TWIST1 ED_50_ values were uncorrelated with SOX9 ED_50_ values (Figure S2B). Thus, despite extensive co-regulation of REs by both TFs, dosage sensitivity to individual TFs differs at shared targets.

We next defined predictive tasks for the full depletion effect and ED_50_ considering previously observed correlates. To predict the full depletion effect, we used all 151,457 REs. To define the set of REs for the ED_50_ prediction task, we considered previously observed correlates of ED_50_. We previously observed that REs likely directly regulated by SOX9 (defined as losing accessibility within 3 h of full SOX9 depletion) showed substantially higher ED_50_ than secondary effects mediated by other downstream TFs.^11^ Of the 9,279 direct SOX9 targets defined this way, 4,226 (46%) have an ED_50_ < 30 (buffered), while 5,013 (54%) have an ED_50_ > 30 (sensitive). Defining direct SOX9 targets by chromatin immunoprecipitation followed by sequencing (ChIP-seq; previously only analyzed aggregating over many regions) yielded 2,565 (65%) buffered and 1,375 (35%) sensitive targets. We observed a similar phenomenon for TWIST1 sensitivity, where putative direct targets (here defined as TWIST1 bound by ChIP-seq and downregulated upon full depletion) showed higher ED_50_ values compared to other TWIST1-dependent REs (Figure S2C), a difference that could not be explained by differences in unperturbed accessibility levels between direct and indirect targets (Figures S2D and S2E). Of the 21,172 direct TWIST1 targets defined by ChIP-seq, 5,452 (26%) have an ED_50_ < 30 (buffered), while 15,720 (73%) have an ED_50_ > 30 (sensitive). Alternatively, defining TWIST1 direct targets in the same way as we originally did for SOX9 (by accessibility loss within 3 h of full depletion) yielded 6,124 (23%) buffered and 20,442 (77%) sensitive targets. Finally, 13,905 REs show TWIST1 binding by ChIP-seq but are not TWIST1 dependent, while 3,103 REs show SOX9 binding but are not SOX9 dependent. Such REs may contain artifactual ChIP-seq signals, may take longer than 24–48 h to show accessibility changes, may be sufficiently bound by other TFs such that SOX9/TWIST1 regulation is not important for accessibility, or may be impacted through mechanisms other than accessibility. Moving forward, we sought to predict the ED_50_ among the 21,172 REs directly regulated by TWIST1 as defined by ChIP-seq and among the 9,279 REs directly regulated by SOX9 as defined by 3 h accessibility loss.

Our transfer learning approach involves pretraining a deep learning model to predict chromatin accessibility levels among ATAC peaks and matched background regions in unperturbed cells, followed by model fine-tuning to predict either the full depletion effect or ED_50_ for TWIST1 or SOX9 (four prediction tasks) among the above-defined sets of REs (Figure 1E). We used a convolutional neural network (CNN) architecture from the recently developed ChromBPNet model, which provides quantitative predictions for ∼1 kb regions based on a 2,114 bp receptive field (see STAR Methods) and was explicitly designed to account for transposase insertion bias in ATAC-seq.^22^ For baseline comparisons, we used regularized (least absolute shrinkage and selection operator [LASSO]) linear or random forest regression with standard position weight matrix (PWM) matching of all known motifs as predictors. Because unperturbed chromatin accessibility was correlated with both the full depletion effect and ED_50_ (Figure S2F), we also included this as a predictor in the LASSO and random forest models. The same training-test-validation splits were used for pretraining, fine-tuning, and the baseline approaches. Both pretraining and fine-tuning were only done on the designated training set. We benchmarked all predictions against a lower-bound estimate of experimental reproducibility obtained by comparing the full depletion effect or ED_50_ estimates between two halves of biological replicates.

**Figure 2:**
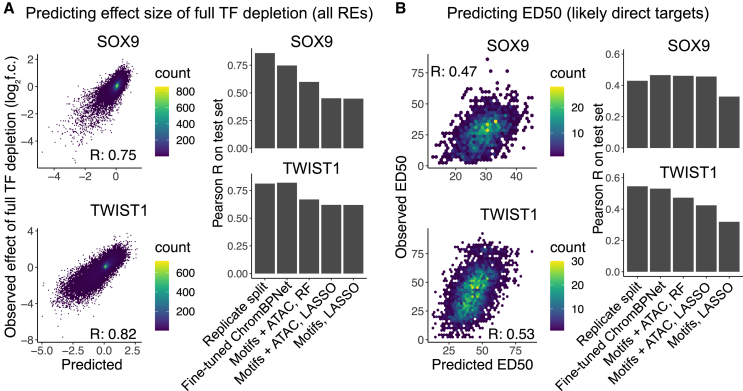
Accurate prediction of the RE response to SOX9 and TWIST1 dosage by transfer learning (A) Performance of fine-tuned ChromBPNet model (left) on effect of full depletion of SOX9 (top) or TWIST1 (bottom) compared to lower-bound estimate of experimental reproducibility (replicate split) and baseline approaches (right). (B) Same plots as in (A) but for ED_50_, only considering likely direct targets of SOX9 or TWIST1. See also Figures S2 and S3.

Fine-tuned ChromBPNet models substantially outperformed baseline approaches and showed prediction accuracy roughly on par with experimental reproducibility (Figures 2A and 2B). In three of the four prediction tasks (not for SOX9 ED_50_ prediction), fine-tuned ChromBPNet also outperformed baseline approaches that included real data (chromatin accessibility in unperturbed cells) as a predictor. Omitting either the pretraining or fine-tuning steps resulted in a substantial drop in accuracy (Figure S3A). Prediction accuracy was stable across replicate training runs and independent training-validation-testing data splits (Figure S3B). While the absolute predictive accuracy of the full depletion effects was ∼55%–60% higher than that of ED_50_, this is driven by two technical factors. First, the full depletion predictions are over all 151,457 REs, whereas ED_50_ predictions are for the smaller subset of likely direct targets of SOX9 and TWIST1. Indeed, when subsetting the full depletion effect predictions to these likely direct targets, the accuracy improvement over ED_50_ predictions is lower (∼15%–43%; Figure S3C). Second, the full depletion estimate is less noisy than the ED_50_ estimate, as indicated by the higher correlation between replicate splits (Figures 2A and 2B). Nonetheless, these results indicate that transfer learning can accurately predict both the magnitude (effect of complete TF loss) and shape (ED_50_) of the quantitative response to TF dosage.

### Sequence features predictive of RE responsiveness to TF dosage

We next interpreted the fine-tuned deep learning models to discover sequence features that underlie their improved performance. We used DeepLIFT^23^ to generate contribution scores, which quantify how each RE base pair contributes to the predicted full depletion effect or ED_50_. We used TF motif discovery from importance scores (TF-MoDISco)^24^ to discover motifs with high contribution scores, summarized as contribution weight matrices (CWMs). We first assessed sequence features predictive of the full depletion effect across all REs (both directly and indirectly regulated). Here, sequence features with a negative predictive contribution to the log_2_ fold change upon full depletion predict loss of accessibility, while those with a positive predictive contribution predict gains of accessibility. We and others have previously shown that SOX9 binds a palindrome motif with 3–5 bp spacing, while TWIST1 cooperatively binds the composite Coordinator motif, consisting of an E-box (CANNTG) contacted directly by TWIST1 and homeobox (TAATT[A/G]) sequences separated by an A-rich spacer and bound by various homeodomain-containing TFs.^11^^,^^14^^,^^25^ For SOX9, we found that the 3–5 bp palindrome motifs all predict loss of accessibility following full depletion (Figure S4A). For TWIST1, both canonical and variant instances of Coordinator predict a larger loss of accessibility (Figure S4B). We expand on these variant motifs further in the analysis of sequence features predictive of ED_50_. Motifs for other specific TFs (TFAP2, TWIST1, and JUN/FOS for SOX9 and TFAP2, SIX, and NR2F for TWIST1) predicted gains of accessibility following full depletion, consistent with our previous observations of secondary effects following SOX9 or TWIST1 depletion. E-box motifs specific to the repressive TFs SNAI1/2 (CAGGTG) were also predictive of losses in accessibility, mostly for REs that showed delayed effects following SOX9 depletion (Figure S4A).

**Figure 3:**
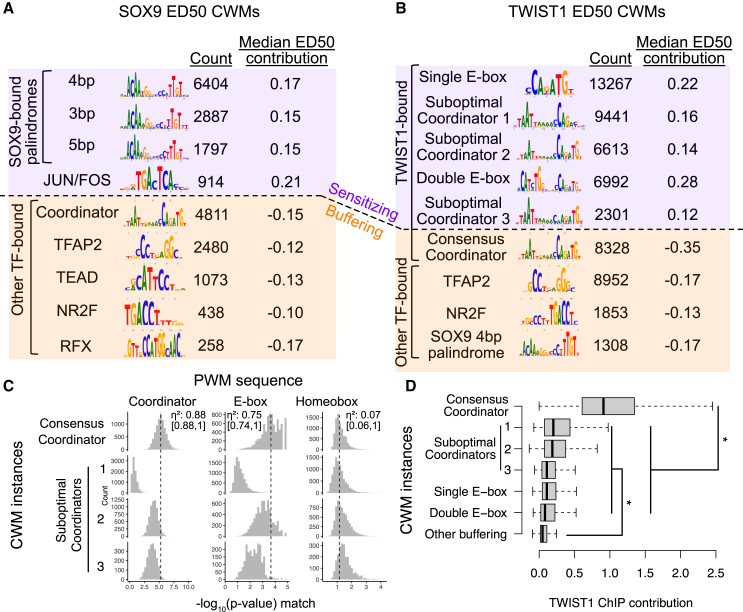
Sequence features predictive of RE sensitivity to SOX9 or TWIST1 dosage (A and B) Top contribution weight matrices (CWMs) predictive of (A) SOX9 or (B) TWIST1 ED_50_. Number of individual occurrences of each CWM is indicated under the “count” column, as well as the median of the mean base-pair-level contribution scores at all occurrences. “Sensitizing” refers to positive ED_50_ contributions, whereas “buffering” indicates negative ED_50_ contributions. (C) For all individual instances of the indicated CWMs predictive of TWIST1 ED_50_ (rows, i.e., from B), the strength of that CWM’s sequence match to the given position weight matrix (PWM; columns) is shown (x axis). The dotted line indicates the median match strength (−log10 *p* value) of the consensus Coordinator CWM (top row) to the indicated PWM. η^2^ and 95% confidence interval in brackets are from ANOVA of −log10(PWM match) as a function of TWIST1 CWM type. (D) For the same CWMs, the distribution of contribution to TWIST1 binding, estimated from training a BPNet model on TWIST1 ChIP-seq, is shown. See also Figures S4 and S5. *n* of groups from top to bottom: 13,528, 5,098, 10,109, 1,869, 5,393, 7,745, 7,902. ∗*p* < 2.2e−16, two-sided Wilcoxon rank-sum test between each pair of the two groups indicated.

We next focused on sequence features predictive of ED_50_ among the likely direct targets of each TF. Here, sequence features that have a negative predictive contribution to the ED_50_ predict buffering, while those with a positive predictive contribution predict sensitivity. For both SOX9 and TWIST1, motifs for TFs other than the perturbed one were predictive of a more buffered response (lower ED_50_), with the exception of the JUN/FOS motif, which was predictive of sensitivity to SOX9 dosage (Figures 3A and 3B). In contrast, motifs bound solely by the perturbed TF, such as the SOX9 palindrome and the single or double E-box, were predictive of increased sensitivity (higher ED_50_) (Figures 3A and 3B). Systematically comparing ED_50_ contribution scores between homotypic and heterotypic motifs for TWIST1 and SOX9 showed that homotypic motifs had significantly higher, positive ED_50_ contributions, while heterotypic motifs had negative contributions (Figure S4C). The observation that buffering is associated with the presence of other TF motifs and that sensitivity is linked to the homotypic motifs for the perturbed TF recapitulates the patterns we previously observed when analyzing SOX9 ED_50_ and extends them to be predictive of dosage responses to TWIST1. Binding by paralogous and potentially redundant TFs (SOX8/10 for SOX9 and HAND1/2 for TWIST1) is unlikely to explain buffered RE responses, as SOX8/10 and HAND1/2 are very lowly expressed in unperturbed CNCCs and do not increase in expression upon SOX9 depletion (Figure S4D).

Surprisingly, however, for TWIST1, diverse types of Coordinator motifs (which are bound by TWIST1 in the E-box portion and homeodomain TFs in the homeobox portion) showed opposing effects on ED_50_—CWMs with high similarity to the consensus Coordinator were strongly predictive of buffering, whereas degenerate CWMs lacking preferred nucleotides were predictive of sensitivity. These degenerate CWMs fell into three subclusters: a degenerate sequence at the (1) final two or (2) first two base pairs of the Coordinator E-box (CANNNN and NNNNTG) or (3) substitutions throughout the motif (Figure 3B). We have previously found, using electrophoretic mobility shift assays (EMSAs), that TWIST1 binds the suboptimal E-box and Coordinator variants containing a single base-pair substitution with ∼3- to 5-fold lower affinity than the canonical Coordinator motif.^14^ The Coordinator variants we discovered here have even more degeneracy than the single base-pair substitutions previously tested in EMSAs, suggesting that even very-low-affinity motifs can contribute substantially to TWIST1 responsiveness and confer sensitivity.

To further substantiate low-affinity binding site contributions, we quantified how individual instances of sensitizing or buffering CWMs match their canonical corresponding PWMs and predict TWIST1 binding. We adapted a previously developed method^6^ to identify individual occurrences of highly contributing sequences independent of their specific identity and then matched them to a specific CWM among those discovered as globally predictive of sensitivity or buffering. We then scanned each CWM occurrence identified in this way with the corresponding PWM model (Tables S3 and S4). Most single or double E-box occurrences sensitizing for TWIST1 showed weak but detectable matches (Figure 3C). The Coordinator CWM instances showed the most stark differences, with most (55.3%) of the sensitizing Coordinator instances undetectable even by lenient PWM matching thresholds, whereas almost all (92.8%) buffering Coordinator instances show strong PWM matches (Figure 3C). We then assessed how individual CWM instances contributed to TWIST1 or SOX9 binding, as measured by applying BPNet^7^ to ChIP-seq of each TF. The buffering Coordinator instances were highly predictive of TWIST1 binding, while the degenerate, sensitizing Coordinator instances (as well as the single and double E-boxes) showed less predictive contributions on average but significantly more than other CWMs (Figure 3D). For SOX9, the palindrome sequences showed strong PWM matches and were similar to each other in predicting binding, with slightly higher predictive power for the 4 bp palindrome (Figures S4D and S4E).

Given that we used direct targets defined differently for SOX9 (rapid accessibility loss after 3 h depletion) versus TWIST1 (bound by ChIP-seq), we repeated analyses using the alternative definition for each TF (i.e., bound by ChIP-seq to define SOX9 direct targets and rapid accessibility loss after 3 h depletion to define TWIST1 direct targets). We found very similar sequence features predictive of TWIST1 or SOX9 ED_50_ with direct targets defined in this way. TWIST1 or SOX9 ED_50_ contribution scores of individual CWM instances were highly correlated between models that defined direct targets by either of the two approaches (Figures S5A–S5C). Together, these results indicate that the chromatin response to TF dosage consists of TF-shared logic, where heterotypic co-binding with other TFs predicts buffered responses for both SOX9 and TWIST1, as well as TF-specific logic, where low-affinity TWIST1 binding sites are predictive of sensitivity but high-affinity sites are buffering.

### Buffering and sensitizing motif occurrences show distinct regulatory logic but are similarly constrained

**Figure 4:**
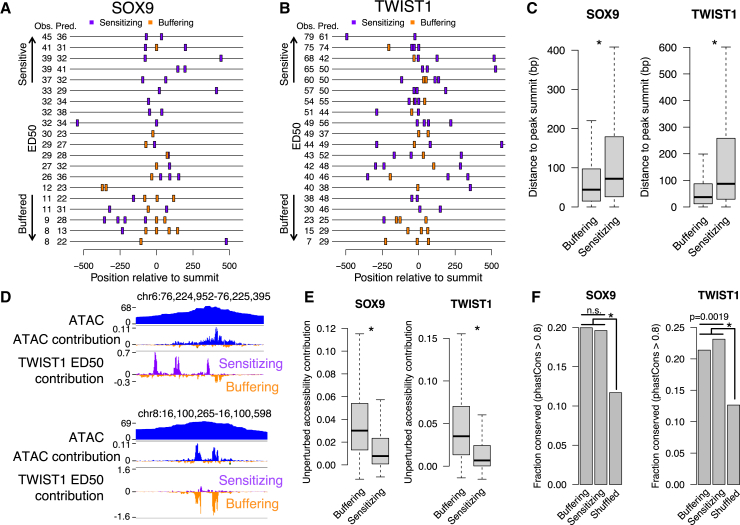
Distinct and shared features of sensitizing and buffering sequences (A and B) 15 randomly sampled SOX9 (A) or TWIST1 (B) target REs with buffering sensitizing motif occurrences (colors) indicated. (C) Distance to ATAC peak summit of all sensitizing or buffering motif occurrences for SOX9 (left) or TWIST1 (right). (D) Examples of sensitizing and buffering occurrences for TWIST1. (E) Accessibility contributions in unperturbed cells of all sensitizing or buffering motif occurrences for SOX9 (left) or TWIST1 (right). (F) Fraction of buffering, sensitizing, or location-shuffled occurrences showing evidence of evolutionary conservation estimated from primate genome alignments for SOX9 (left) or TWIST1 (right). Comparisons in (C) and (E): ∗*p* < 2.2e−16 by two-sided Wilcoxon rank-sum test. Comparisons in (F): n.s., *p* > 0.05 and ∗*p* < 2.2e−16 by two-sided Fisher’s exact test for indicated pairs of groups. *n* for groups in (C), (E), and (F): SOX9 buffering, 9,953; SOX9 sensitizing, 12,716; SOX9 shuffled, 26,596; TWIST1 buffering, 23,647; TWIST1 sensitizing, 38,614; and TWIST1 shuffled, 71,012. See also Figure S6.

We next assessed distinct and shared features of buffering and sensitizing sequences. There were a total of 22,667 buffering or sensitizing motif occurrences for SOX9 (mean: 2.5 per RE) and 62,261 for TWIST1 (mean: 3 per RE) across all likely direct target REs. Most REs contained both sensitizing and buffering motif occurrences (Figures 4A, 4B, S5D, and S5E), indicating that combinations of the two types can concurrently tune the RE dosage response. With respect to positioning, sensitizing motif occurrences were located further away from the RE summit (point of highest accessibility across the RE) as compared to buffering motif occurrences (Figure 4C). We observed similar results when analyzing specific subtypes of sensitizing or buffering motifs (Figures S5F and S5G). We then compared how these buffering/sensitizing motif occurrences contributed to accessibility levels in unperturbed cells as estimated from the pretrained ChromBPNet model. Contribution tracks at individual loci highlighted multiple sensitizing instances that would not be detected in the pretrained model of unperturbed accessibility, in contrast to buffering instances, which tracked with unperturbed accessibility contributions (Figure 4D). Indeed, for both SOX9 and TWIST1, buffering motif occurrences showed strong, positive contributions to unperturbed accessibility, while sensitizing motif occurrences showed weaker contributions (Figure 4E).

Weaker unperturbed accessibility contributions and distributed positioning of sensitizing motif occurrences raises the question of whether they are as biologically relevant as buffering motifs. To assess this, we compared the signatures of selection between buffering and sensitizing motif occurrences, shuffling the position of motif occurrences within the same REs as a control. We considered base-pair-level estimates of negative selection from multiple primate and vertebrate genome alignments, as well as joint estimates combining between-species divergence with human polymorphism data.^26^ Sensitizing and buffering motif occurrences showed similar degrees of negative selection, and both were higher than shuffled motif occurrences (Figures 4F and S6A–S6D). Neither class of motif occurrence showed evidence for positive selection (Figure S6E). These results suggest that low-affinity and homotypic motifs that predict sensitive TF dosage responses contribute to organismal fitness and are thus evolutionarily constrained.

### Enhancer reporter assays validate model predictions on mutant sequences

**Figure 5:**
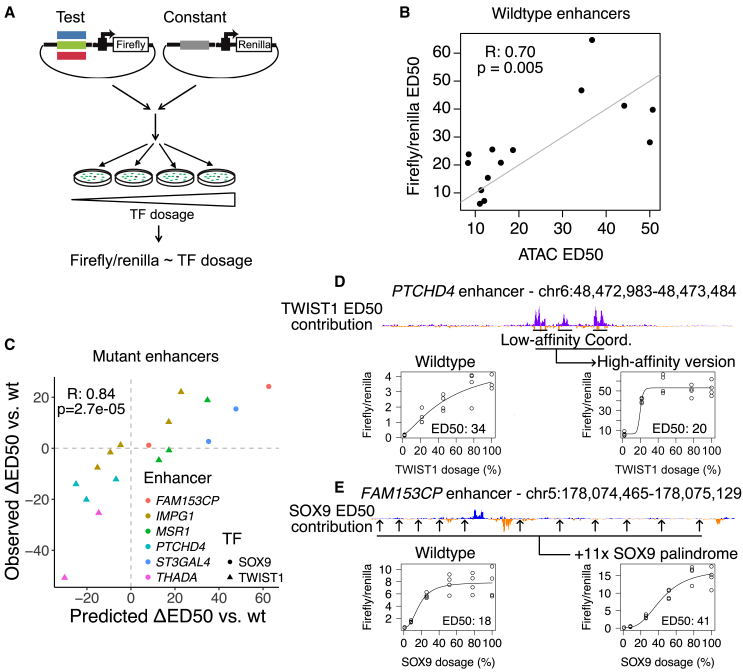
Experimental validation of model predictions by enhancer reporter assays (A) Schematic of enhancer reporter assay approach. (B) Comparison of ED_50_ with respect to SOX9 or TWIST1 dosage measured endogenously with ATAC-seq (x axis) and assessed in the episomal reporter assay (y axis). Each point is a different wild-type enhancer sequence; ED_50_ was assessed with at least 4 replicates. Pearson correlation coefficient (R) and *p* indicated on plot. (C) Predicted (x axis) versus observed (y axis) effect of model-guided mutations on ED_50_ of various enhancers (colors) as a function of TWIST1 or SOX9 dosage (shapes). ED_50_ was assessed with 4 replicates. Pearson correlation coefficient (R) and *p* indicated on plot. (D) Example of PTCHD4 enhancer, where converting three low-affinity Coordinators/single E-box (peaks in the ED_50_ contribution track) to high-affinity Coordinators has a buffering effect. (E) Example of FAM153CP enhancer, where ectopically inserting 11 SOX9 palindromes at the indicated positions has a sensitizing effect as a function of SOX9 dosage. See also Figures S7–S9.

We sought to experimentally validate the sensitizing and buffering sequences revealed by fine-tuned ChromBPNet models. We used enhancer reporter assays, in which an RE is cloned upstream of a minimal promoter and a luciferase reporter gene and assayed for enhancer activity in CNCCs with a distinct TWIST1 or SOX9 dosage achieved using dTAG (Figure 5A). While there are known differences between such assays that test reporter transcriptional activation and the endogenous measures of chromatin accessibility we used to fine-tune ChromBPNet models, previous studies have found RE accessibility to be among the most predictive biochemical features for reporter activity,^27^ and the two assays share broadly similar predictive sequence features.^15^ We chose 19 REs that were strongly TF dependent (12 TWIST1 dependent and 7 SOX9 dependent), located distally from promoters, highly accessible, and positive for H3K27ac (an active enhancer mark). We tested all 19 chosen REs with the enhancer reporter assay, observing a positive correlation between endogenous RE accessibility and reporter activity at 100% TF dosage (Figure S7A; Table S5). For the 15 REs that showed significant enhancer activity at 100% TF dosage, we observed a positive correlation between the ED_50_ of endogenous accessibility and enhancer reporter activity (Figure 5B). We observed a similar correlation for the full depletion effect (Figure S7B). Thus, episomal reporter assays can recapitulate differences in TF responsiveness observed with endogenous chromatin accessibility measurements.

We next tested the effect of sensitizing and buffering features by assaying mutant enhancer sequences predicted to change ED_50_. We selected 7 wild-type enhancers (5 TWIST1 dependent and 2 SOX9 dependent) with a range of ED_50_ values and designed mutant sequences, guided by principles from model interpretation. We designed minimal substitutions to modulate TWIST1 binding site affinity and composition, as well as larger changes scrambling or inserting sensitizing elements at various positions (Table S6; see Figure S8 for schematic). We designed mutant sequences to retain accessibility/enhancer activity such that their ED_50_ could still be assayed, resulting in 18 mutant sequences from the 7 wild-type enhancers. We tested each mutant in the enhancer reporter assay in parallel with its corresponding wild-type sequence, calculating the difference in ED_50_ between the two (ΔED_50_). One mutant enhancer (SOX9 motif disruption in a TWIST1-dependent enhancer at the *RNF157* locus) almost completely lost activity, precluding us from analyzing its ΔED_50_. Overall, this analysis uncovered a strong, positive correlation between the predicted and observed ΔED_50_ values, thus validating the predictive sequence features revealed by the fine-tuned ChromBPNet model (Figure 5C).

As an example, we observed substantial buffering effects with mutants of the *PTCHD4* enhancer, where converting three single E-boxes/low-affinity Coordinator instances to high-affinity Coordinators decreased ED_50_ from 33.9 to 19.8 and increased activity at 100% TWIST1 dosage by ∼14.5-fold (Figure 5D). Conversely, converting two high-affinity Coordinator instances to a double E-box and low-affinity Coordinator in the *MSR1* enhancer increased ED_50_ from 19.1 to 38 and decreased activity by ∼2.5-fold (Figure S9A). However, this correlation between buffering effects of mutations and increases in activity at 100% dosage was not always the case (Figure S9B). For example, the insertion of 5 single E-box instances into the moderately buffered *IMPG1* enhancer increased ED_50_ from 18 to 45.3 with a minimal change in activity at 100% TWIST1 dosage (Figure S9C). These results generalized to SOX9-dependent enhancers, as increasing the ratio of homotypic to heterotypic motifs by inserting 11 SOX9 palindrome instances into the buffered *FAM153CP* enhancer increased ED_50_ from 18 to 41 while increasing activity at 100% SOX9 dosage by ∼2-fold (Figure 5E). Sensitivity to TF dosage is, therefore, not a simple correlate of unperturbed accessibility or enhancer activity, with a partially distinct sequence logic that can be experimentally modulated in a model-guided fashion.

### TF-nucleosome competition can explain the sensitizing effect of low-affinity sites

We sought to build biophysical intuition for why, as observed for TWIST1, low-affinity sites have a sensitizing effect when added in the vicinity of high-affinity, buffering sites. We considered a previously developed model^28^ in which competition between TFs and nucleosomes for binding to DNA (TF-nucleosome competition) induces TF cooperativity, referred to as “nucleosome-mediated cooperativity.” TF-nucleosome competition through the suppression of TF binding to nucleosome-bound DNA is controlled by a single parameter *c*, defined as the ratio of TF motif affinities between the nucleosome-free and -bound states. Given TF and nucleosome binding constants (the latter estimated from experimental data^28^), the model allows for derivation of steady-state RE accessibility (inverse of nucleosome occupancy) as a function of TF concentration using a statistical mechanics approach.

**Figure 6:**
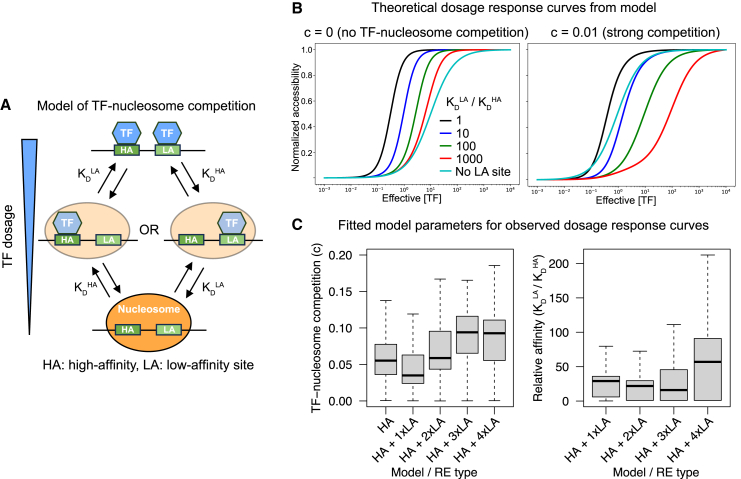
TF-nucleosome competition can explain the sensitizing effect of low-affinity sites (A) Schematic of model of TF-nucleosome competition originally proposed by Mirny.^28^ (B) Effect of low-affinity site (colors) or no site (gray) on theoretical dosage curves under model with no TF-nucleosome competition (left) or strong TF-nucleosome competition (right). The sensitizing effect of low-affinity sites (shift to right relative to gray curve) is only observed with competition. (C) Values of *c* (left) or low-/high-affinity site K_D_ obtained by fitting model to observed dosage response curves for REs containing only high-affinity Coordinator sites (HA) or a mix of high-affinity sites and the indicated number of low-affinity Coordinator sites (HA + 1/2/3xLA). *n* from left to right in left plot: 592, 925, 525, and 360 and from left to right in right plot: 925, 525, and 360. See also Figures S10 and S11.

We implemented a simple case of this model (Figure 6A) with one high-affinity site, varied the K_D_ of a second site such that it went from high affinity to low affinity to non-existent (higher K_D_ meaning lower affinity), and calculated accessibility dosage-response curves under varying degrees of TF-nucleosome competition by tuning *c*. We found that without TF-nucleosome competition (*c* = 0), the addition of any second site (high or low affinity) resulted in a more buffered dosage response (lower ED_50_) (Figure 6B), in contrast to the effects we observed from the ChromBPNet model and validated with enhancer reporter assays. In contrast, under strong competition (*c* = 0.01, i.e., TF binding has 100-fold lower affinity in the nucleosome-bound state), the addition of a second site with at least ∼10-fold higher K_D_ than the first site increases ED_50_ (Figure 6B), replicating the sensitizing effect we previously observed. With weak TF-nucleosome competition (*c* = 0.001), the second site had to be relatively weaker (∼1,000-fold) in order to have a sensitizing effect, but the results were qualitatively similar (Figure S10A).

We next assessed which values of *c* and relative site affinities were best supported by our data. We fit the model of TF-nucleosome competition to dosage-response curves (parametrized by the ED_50_ and Hill coefficient) of 540 REs only containing high-affinity, buffering Coordinator motifs. We obtained the best fits with an effective numbers of sites (*n*) ≥3 (Figure S10B), which yielded a median *c* of 0.062 (Figure 6C). We then held the effective number of high-affinity sites fixed at 3 and fit four types of models, each with one to three additional low-affinity sites, based on the number of observed low-affinity (sensitizing) sites in the RE (total: 1,810 REs). The median *c* value of each model type ranged from 0.033 to 0.087, and the median K_D_ ratio between the low- and high-affinity sites ranged from ∼13- to ∼29-fold (Figure 6C). These fitted values support the theoretical scenario of strong TF-nucleosome competition and are of the same order of magnitude as the ≥5-fold affinity difference between canonical and degenerate Coordinator sequences in our previous EMSA results.^14^ We obtained similar fitted values when fixing the number of low-affinity sites in the model (Figure S10C).

To assess whether heterotypic co-binding by other TFs, as observed for both TWIST1 and SOX9, also required TF-nucleosome competition, we adapted the model to include a single site for a second, unperturbed TF. Analysis of this model revealed that when the unperturbed TF had an effective concentration similar to or larger than the perturbed TF, it resulted in a more buffered dosage response, even in the absence of TF-nucleosome competition (*c* = 0, Figure S11A); this effect was slightly reduced under strong competition (*c* = 0.01, Figure S11B). We also fit this model to experimental SOX9 dosage-response curves for REs containing only one SOX9 motif (1,372 REs), one additional Coordinator motif (686 REs), or one additional TFAP2 motif (386 REs), obtaining substantially lower *c* values (<0.005) than observed for high- and low-affinity Coordinator motifs at TWIST1-dependent REs (Figure S11C). Thus, TF-nucleosome competition is not necessary to explain the buffering effect of heterotypic TF motifs.

To complement our modeling approach, we sought to assess how nucleosome occupancy and positioning change as a function of TWIST1 dosage relative to individual occurrences of high- and low-affinity TWIST1 motifs. We focused on TWIST1 because TF-nucleosome competition was not necessary to explain the buffering effect of heterotypic motifs as observed for SOX9. We used NucleoATAC^29^ to infer nucleosome occupancy and position from our ATAC-seq data at different TWIST1 dosages, aggregating analyses around all high- (canonical Coordinator) or low- (degenerate Coordinator or E-box) affinity motifs. At 100% TWIST1 dosage, high-affinity motifs showed a greater reduction in inferred nucleosome occupancy in the surrounding 150 bp than low-affinity motifs. Furthermore, high-affinity motifs did not show large aggregate changes in overlapping nucleosome occupancy until ∼0% TWIST1 dosage, in contrast to low-affinity motifs, which showed clear increases in occupancy at ∼22% TWIST1 dosage (Figure S11D). The difference in nucleosome positioning at 100% TWIST1 dosage was partially driven by differing positions within REs, as low-affinity motifs located in the central 100 bp of their REs showed more similar (but still slightly increased) nucleosome occupancy in the surrounding 150 bp relative to central high-affinity motifs. Importantly, however, the differences in sensitivity to nucleosome occlusion at ∼22% TWIST1 dosage held regardless of whether central or peripheral motifs were analyzed (Figure S11E). This indicates that both high- and low-affinity motifs compete against nucleosomes for DNA occupancy, with high-affinity motifs doing so more effectively than low-affinity motifs. Together, our findings suggest that TF-nucleosome competition can explain the sensitizing effect of low-affinity sites when added to REs containing high-affinity sites.

## Discussion

Here, we have used transfer learning to reveal the sequence features underlying the RE response to SOX9 or TWIST1 dosage. Our study adds to the growing body of work demonstrating the importance of low-affinity TF binding sites for precise control of RE activity and gene expression. Low-affinity binding by TFs has been shown to confer specificity in both developmental^30^^,^^31^ and synthetic^32^ systems, and increases in the affinity of such sites can result in ectopic expression and developmental phenotypes.^33^^,^^34^ Our results broadly agree with these studies, as REs consisting solely of low-affinity TWIST1 binding sites have higher ED_50_ and require higher concentrations of TWIST1 to be active. Our observations further indicate that low-affinity sites play an important role in setting the shape of the TF dosage-response curve even in REs with other, high-affinity sites.

We found low-affinity TWIST1 sites to be important for TWIST1 dosage response but did not identify low-affinity (i.e., single) SOX9 sites as important for sensitivity to SOX9 dosage. This could be due to the fewer training examples for SOX9 sensitivity, but the fact that single SOX sites were not discovered as predictive of the SOX9 full depletion effect, which uses many more training examples, suggests that such data limitation is an unlikely explanation. Alternatively, single SOX motifs in CNCCs may be of such low affinity that they effectively function as having no binding site at all. Indeed, *in vitro* assays showed highly cooperative SOX9 binding to the 3–5 bp palindrome, such that binding to one site enhanced binding to the second by >100-fold.^25^ The only other motifs predictive of SOX9 dosage sensitivity were for the AP-1 TFs (dimers of the JUN, FOS, or ATF proteins). Given that SOX9 and AP-1 physically interact and bind overlapping genomic regions in chondrocytes,^35^ AP-1 sites may function as low-affinity SOX9 sites by indirectly recruiting SOX9, but further experiments are needed to test this hypothesis.

Using a previously proposed model of TF-nucleosome competition, we showed that such competition is sufficient to explain the sensitizing effect of low-affinity binding sites. This model assumes a distance of up to ∼150–200 bp (the approximate DNA length of a single nucleosome) between low- and high-affinity binding sites, an assumption that holds true for a plurality (∼40%) of the low-affinity sites studied here, with the majority of the remaining sites located within one or two nucleosomal lengths (Figure S8D). The 150–200 bp range of the TF-nucleosome competition model could be increased beyond a single nucleosome by inter-nucleosomal interactions^36^ or TF-mediated recruitment of chromatin modifiers. Indeed, a recent study of single-molecule TFs and nucleosome occupancy added out-of-equilibrium kinetics (such as those induced by modifier recruitment) to explain the observed TF co-binding as a function of nucleosome occupancy.^37^ Explicitly modeling the effect of multi-nucleosome conformations and/or chromatin modifier recruitment on accessibility dosage-response curves therefore represents an important future avenue.

Our results also provide a potential explanation for phenotypic specificity associated with TF dosage perturbations in human traits and diseases, despite widespread TF co-binding at REs. We observed that dosage-sensitive REs are largely non-overlapping between TWIST1 and SOX9, even among likely direct targets of both TFs. In this scenario, quantitative reductions in TWIST1 or SOX9 dosage preferentially impact largely distinct sets of REs, which in turn may regulate distinct genes and ultimately downstream phenotypes. In effect, these results extend our previously proposed model of phenotypic specificity at distinct SOX9 dosages^11^ to also explain how distinct phenotypes can arise from dosage perturbations of different TFs active in the same cell type.

Our study highlights the power of applying deep learning to epigenomic data gathered from perturbed states as a means of learning important *cis*-regulatory logic that would not be apparent in steady-state data. Many of the low-affinity, sensitizing sequences that our approach discovered would not be detected by traditional motif-matching approaches or deep learning models of unperturbed chromatin accessibility. This additional layer of the *cis*-regulatory code, one that sets the response to TF dosage changes, could be modulated by trait-associated sequence variants discovered in genome-wide association studies (GWASs). The types of models we have described here could, therefore, ultimately aid in interpreting and fine-mapping GWAS variants, particularly those that cannot readily be explained by effects on chromatin state in an unperturbed setting.

### Limitations of the study

While we focused on TF-nucleosome competition and motif-driven interactions as key mechanisms, other regulatory factors, such as chromatin remodelers or co-factor recruitment, were not explicitly modeled and may contribute to unexplained variance in TF dosage responses. We experimentally validated model predictions using enhancer reporter assays; although we demonstrated that such episomal assays recapitulate differences in TF responsiveness observed from endogenous chromatin accessibility, it is possible that additional features present only in endogenous contexts influence the observed dosage-response relationships. We studied two TFs (TWIST1 and SOX9) in a single cell type (hESC-derived CNCCs); therefore, the generalizability of our findings to other cell types and a broader range of TFs remains unclear.

## Resource availability

### Lead contact

Further information and requests for resources and reagents should be directed to and will be fulfilled by the lead contact, Sahin Naqvi (sahin.naqvi@childrens.harvard.edu).

### Materials availability

The plasmids generated in this study are available in Addgene with the indicated plasmid numbers listed in the key resources table. All other reagents are available upon request to Sahin Naqvi.

### Data and code availability

The raw sequencing files generated during this study are available on the Gene Expression Omnibus (GEO: GSE267008); corresponding processed data and trained deep learning models are available on Zenodo^38^ (Zenodo: https://doi.org/10.5281/zenodo.14633030). The ChromBPNet package is available on GitHub (Github: https://github.com/kundajelab/chrombpnet). Additional code used for sequencing data analysis and fine-tuning ChromBPNet models, as well as the output of pretrained and fine-tuned ChromBPNet models, is available on Zenodo.^38^

## Acknowledgments

We thank Surag Nair, Jordan Valgardson, and Tony Zeng for advice on the transfer learning approach. This work was supported by a Helen Hay Whitney Foundation Fellowship and 10.13039/100000002NIH grants K99 DE032729 and R00 DE032729 to S.N.; an HHMI-Damon Runyon Cancer Research Foundation fellowship (DRG-2420-21) to S.K.; the Stanford Graduate Fellowship and the NSF Graduate Research Fellowship (DGE-2146755) to S.T.; NIH grant R01 HG008140 to J.K.P.; and NIH grant R35 GM131757, the 10.13039/501100008483NOMIS Foundation, funding from the 10.13039/100000011Howard Hughes Medical Institute, a Lorry Lokey endowed professorship, and a Stinehart Reed award to J.W. A.P. was supported by the Stanford Bio-X Fellowship.

## Author contributions

Conceptualization, S.N., J.K.P., and J.W.; methodology, S.N., S.K., and S.T.; software, A.P. and A.K.; formal analysis, S.N. and S.T.; investigation, S.N. and S.K.; resources, S.N., A.K., J.K.P., and J.W.; writing – original draft, S.N. and J.W.; writing – review & editing, S.N., S.K., S.T., J.K.P., and J.W.; visualization, S.N. and S.T.; supervision, S.N., J.K.P., and J.W.; project administration, S.N., J.K.P., and J.W.; funding acquisition, S.N., A.K., J.K.P., and J.W.

## Declaration of interests

J.W. is a paid scientific advisory board member at Camp4. A.K. is on the scientific advisory boards of PatchBio, SerImmune, AINovo, TensorBio, and OpenTargets, was a consultant with Illumina, and owns shares in Illumina, Deep Genomics, Immunai, and Freenome, Inc. J.W. is an advisory board member at Cell Press journals, including *Cell*, *Molecular Cell*, and *Developmental Cell*.

## Declaration of generative AI and AI-assisted technologies in the writing process

During the preparation of this work, the authors used ChatGPT (GPT-4o) and Claude (3.5 Sonnet) to shorten specific paragraphs and the summary of the manuscript. ChatGPT and Claude were also used to generate drafts of the highlights and eTOC. After using these tools, the authors reviewed and edited the content as needed and take full responsibility for the content of the publication.

## STAR★Methods

### Key resources table

**Table:** 

REAGENT or RESOURCE	SOURCE	IDENTIFIER
**Antibodies**		

Mouse monoclonal anti-V5	Abcam	Cat# ab27671; RRID:AB_471093

**Chemicals, peptides, and recombinant proteins**		

dTAGV-1	Tocris	Cat# 6914
mTeSR	Stem Cell Technologies	Cat# 85850
Matrigel Growth Factor Reduced (GFR) Basement Membrane Matrix	Corning	Cat# 356231
ReLeSR	Stem Cell Technologies	Cat# 05872
Collagenase IV	GIBCO	Cat# 17104019
DMEM/F12 1:1 medium, with L-glutamine; without HEPES	GE Healthcare	Cat# SH30271.FS
Neurobasal Medium	Thermo Fisher Scientific	Cat# 21103049
Gem21 NeuroPlex Supplement With Vitamin A	Gemini Bio-Products	Cat# 400-160
N2 NeuroPlex Supplement	Gemini Bio-Products	Cat# 400-163
Antibiotic-Antimycotic (100X)	GIBCO	Cat# 15240062
GlutaMAX Supplement (100X)	Life Technologies	Cat# 35050061
Recombinant Human FGF-basic (154 a.a.)	PeproTech	Cat# 100-18B
Animal-Free Recombinant Human EGF	PeproTech	Cat# AF-100-15
Bovine Insulin Powder	Gemini	Cat# 700-112P
Human Plasma Fibronectin Purified Protein	MilliporeSigma	Cat# FC01010MG
Accutase	Sigma-Aldrich	Cat# A6964-100ML
Bovine Serum Albumin (BSA), Fraction V—Serum Replacement Grade	Gemini Bio-Products	Cat# 700-104P
Recombinant Human/Murine/Rat BMP-2 (E.coli derived)	PeproTech	Cat# 120-02
CHIR-99021 (CT99021) HCl	Selleck Chemicals	Cat# S2924
DMEM/High glucose with L-glutamine, sodium pyruvate	Cytiva (formerly GE Healthcare)	Cat# SH30243.01

**Critical commercial assays**		

Dual-Glo Luciferase Assay System	Promega	Cat# E2940
TRIzol Reagent	Invitrogen	Cat# 15596018
FuGENE 6	Promega	Cat# E2691
AMPure XP	Beckman Coulter	Cat# A63881
Qubit dsDNA HS Assay Kit	Invitrogen	Cat# Q32854

**Deposited data**		

ATAC-seq	This paper	GEO: GSE267008
Processed ATAC-seq, trained deep learning models	This paper	Zenodo: https://doi.org/10.5281/zenodo.14633030

**Experimental models: Cell lines**		

Human: Female H9 human embryonic stem cells (hESCs) (wildtype)	WiCell	WA09; RRID: CVCL_9773
SOX9-tagged H9 hESCs	Naqvi et al.^11^	N/A
TWIST1-tagged H9 hESCs	Kim et al.^14^	N/A

**Recombinant DNA**		

Plasmid: pGL3_SFRP1	This paper	Addgene: 232357
Plasmid: pGL3_RNF157	This paper	Addgene: 232358
Plasmid: pGL3_DDX25	This paper	Addgene: 232359
Plasmid: pGL3_LRFN2	This paper	Addgene: 232360
Plasmid: pGL3_SATB2	This paper	Addgene: 232361
Plasmid: pGL3_IMPG1	This paper	Addgene: 232362
Plasmid: pGL3_CALD1	This paper	Addgene: 232363
Plasmid: pGL3_C8orf76	This paper	Addgene: 232364
Plasmid: pGL3_FRMD4A	This paper	Addgene: 232365
Plasmid: pGL3_RAD51B	This paper	Addgene: 232366
Plasmid: pGL3_chr18q12.3	This paper	Addgene: 232367
Plasmid: pGL3_VPS13B	This paper	Addgene: 232368
Plasmid: pGL3_COL9A2	This paper	Addgene: 232369
Plasmid: pGL3_MECOM	This paper	Addgene: 232370
Plasmid: pGL3_INPP5F	This paper	Addgene: 232371
Plasmid: pGL3_THADA_alltoneg0	This paper	Addgene: 232372
Plasmid: pGL3_RNF157_SOX9scr_neg0str	This paper	Addgene: 232373
Plasmid: pGL3_ST3GAL4	This paper	Addgene: 232374
Plasmid: pGL3_PTCHD4_pos0toneg0	This paper	Addgene: 232375
Plasmid: pGL3_RNF157_SOX9scramble	This paper	Addgene: 232376
Plasmid: pGL3_FAM153CP	This paper	Addgene: 232377
Plasmid: pGL3_PTCHD4_pos1toneg0	This paper	Addgene: 232378
Plasmid: pGL3_RNF157_neg0SOX9scramble	This paper	Addgene: 232379
Plasmid: pGL3_PTCHD4_pos1pos2pos0toneg0	This paper	Addgene: 232380
Plasmid: pGL3_RNF157_wt	This paper	Addgene: 232381
Plasmid: pGL3_PTCHD4_pos2pos0pos1scramble	This paper	Addgene: 232382
Plasmid: pGL3_MSR1_bothneg0scramble	This paper	Addgene: 232383
Plasmid: pGL3_PTCHD4_pos2toneg0	This paper	Addgene: 232384
Plasmid: pGL3_MSR1_leftneg0topos3	This paper	Addgene: 232385
Plasmid: pGL3_THADA_allscramble	This paper	Addgene: 232386
Plasmid: pGL3_MSR1_bothneg0topos3pos1	This paper	Addgene: 232387
Plasmid: pGL3_THADA_pos1toneg0	This paper	Addgene: 232388
Plasmid: pGL3_MSR1_rightneg0topos1	This paper	Addgene: 232389
Plasmid: pGL3_MYO16	This paper	Addgene: 232390
Plasmid: pGL3_MSR1	This paper	Addgene: 232391
Plasmid: pGL3_PTCHD4	This paper	Addgene: 232392
Plasmid: pGL3_THADA	This paper	Addgene: 232393
Plasmid: pGL3_IMPG1_lpos0scr	This paper	Addgene: 232394
Plasmid: pGL3_IMPG1_lpos0pos1scr	This paper	Addgene: 232395
Plasmid: pGL3_IMPG1_lpos0pos1mpos0scr	This paper	Addgene: 232396
Plasmid: pGL3_IMPG1_pos0x5_1150	This paper	Addgene: 232397
Plasmid: pGL3_IMPG1_pos0x5_1175	This paper	Addgene: 232398
Plasmid: pGL3_ST3GAL4_SOX9repall	This paper	Addgene: 232399
Plasmid: pGL3_FAM153CP_SOX9rep	This paper	Addgene: 232400

**Software and algorithms**		

Benchling	Benchling [Biology Software]. (2017)	https://www.benchling.com/
The R package for Statistical Computing	R Core Team (2019); R version 3.6.0	https://www.r-project.org/
Skewer	Jiang et al.^39^	https://github.com/relipmoc/skewer
sowtie2	Langmead and Salzburg^40^	http://bowtie-bio.sourceforge.net/bowtie2/index.shtml
Samtools	Li et al.^41^	https://www.htslib.org/
Bedtools	Quinlan et al.^42^	https://bedtools.readthedocs.io/en/latest/
R DESeq2 package	Love et al.^43^	https://bioconductor.org/packages/release/bioc/html/DESeq2.html
R edgeR package	Robinson et al.^44^	https://bioconductor.org/packages/release/bioc/html/edgeR.html
R drc package	Ritz et al.^45^	https://cran.r-project.org/web/packages/drc/index.html
ChromBPNet	Pampari et al.^22^	https://github.com/kundajelab/chrombpnet
HOCOMOCO	Kulakovskiy et al.^46^	https://hocomoco11.autosome.org/
Python MOODS package	Korhonen et al.^47^	https://github.com/jhkorhonen/MOODS
R randomForest package	CRAN	https://cran.r-project.org/web/packages/randomForest/index.html
TF-MoDISCO	Shrikumar et al.^24^	https://github.com/kundajelab/tfmodisco
BPNet	Avsec et al.^6^	https://github.com/kundajelab/bpnet
PhastCons and PhyloP scores from 30 primate genomes	UCSC	https://hgdownload.soe.ucsc.edu/goldenPath/hg38/phastCons30way/; https://hgdownload.soe.ucsc.edu/goldenPath/hg38/phyloP30way/
PhastCons and PhyloP scores from 100 primate genomes	UCSC	https://hgdownload.soe.ucsc.edu/goldenPath/hg38/phastCons100way/; https://hgdownload.soe.ucsc.edu/goldenPath/hg38/phyloP100way/
INSIGHT	Gronau et al.^26^	https://compgen.cshl.edu/INSIGHT/
NucleoATAC	Schep et al.^29^	https://github.com/GreenleafLab/NucleoATAC

### Experimental model and study participant details

Female H9 (WA09; RRID: CVCL_9773) hESCs were obtained from WiCell and cultured in either mTeSR1 (Stem Cell Technologies 85850) for at least one passage before differentiation into CNCCs or mTeSR Plus (Stem Cell Technologies 100–0276) for gene editing, single-cell cloning, expansion and maintenance. hESCs were grown on Matrigel growth factor reduced basement membrane matrix (Corning 354230) at 37°C. hESCs were fed every day for mTeSR1 or every 2 days for mTeSR Plus and passaged every 5–6 days using ReLeSR (Stem Cell Technologies 05872).

### Method details

#### Differentiation of hESCs to CNCCs

hESCs were grown for 5–6 days until large colonies formed, and then they were disaggregated using collagenase IV and gentle pipetting. Clumps of about 200 hESCs were washed in PBS and transferred to a 10 cm Petri dish in neural crest differentiation medium (1:1 ratio of DMEM-F12 and Neurobasal, 0.5× Gem21 NeuroPlex supplement with vitamin A (Gemini, 400-160), 0.5× N2 NeuroPlex supplement (Gemini, 400-163), 1× antibiotic–antimycotic, 0.5× Glutamax, 20 ng mL^−1^ bFGF (PeproTech, 100-18B), 20 ng mL^−1^ EGF (PeproTech, AF-100-15) and 5 μg mL^−1^ bovine insulin (Gemini Bio-Products, 700-112P)). After 7–8 days, neural crest emerged from neural spheres attached to the Petri dish, and after 11 days, neural crest cells were passaged onto fibronectin-coated 6-well plates (about 1 million cells per well) using Accutase (Sigma-Aldrich A6964) and fed with neural crest maintenance medium (1:1 ratio of DMEM-F12 and neurobasal, 0.5× Gem21 NeuroPlex supplement with vitamin A (Gemini, 400-160), 0.5× N2 NeuroPlex supplement (Gemini, 400-163), 1× antibiotic–antimycotic, 0.5× Glutamax, 20 ng mL^−1^ bFGF, 20 ng mL^−1^ bFGF EGF and 1 mg mL^−1^ BSA (Gemini)). After 2–3 days, neural crest cells were plated at about 1 million cells per well of a 6-well plate, and the following day cells were fed with neural crest long-term medium (neural crest maintenance medium + 50 pg mL^−1^ BMP2 (PeproTech, 120-02) + 3 μM CHIR-99021 (Selleck Chemicals, S2924; BCh medium)). After transition to BCh medium, CNCCs at subsequent passages were plated at about 800,000 cells per well of a 6-well plate. CNCCs were then passaged twice to passage 4, at which depletion experiments were carried out. For depletion experiments, dTAG^V^-1 (Tocris, 6914) at a range of concentrations was added to BCh medium, with an equivalent amount of dimethyl sulfoxide (DMSO) as vehicle control.

#### Flow cytometry

CNCCs were collected for intracellular staining and flow cytometry using Accutase treatment for 5 min at 37C then washed twice in PBS. Cells were fixed in 4% Paraformaldehyde in PBS for 10 min at RT, washed twice with PBS, and then permeabilized in 0.1% Triton X-100 in PBS for 10 min at RT. Cells were then resuspended in blocking buffer (1% BSA, 3% donkey serum in PBS) and blocked for 40 min at RT, flicking tubes every 10 min to resuspend settled cells. Fixed, permeabilized, and blocked cells were incubated with primary antibody (V5) diluted 1:100 in blocking buffer and incubated on ice for 60 min with flicking every 10 min, followed by two PBS washes and staining with secondary antibody for 60 min at RT. Cells were finally washed two additional times with PBS prior to flow cytometry analysis, which used to measure V5 staining intensity and/or mNeonGreen fluorescence (for *SOX9*-tagged CNCCs) after excluding doublets and debris based on forward and side scatter (Beckman Coulter Cytoflex). Fluorescence values were summarized per biological replicate using geometric means.

#### ATAC-seq collection and library preparation

CNCCs were incubated with BCh medium containing 200 U ml DNase I (Worthington, LS002007) for 30 min and collected using Accutase. Viable cells were counted using a Countess Automated Cell Counter (Invitrogen), and 50,000 viable cells were pelleted at 500 RCF for 5 min at 4 °C and resuspended in ATAC-resuspension buffer (10 mM Tris-HCl pH 7.4, 10 mM NaCl, 3 mM MgCl_2_ in sterile water) containing 0.1% NP-40, 0.1% Tween 20 and 0.01% digitonin and incubated on ice for 3 min. Following wash-out with cold ATAC-resuspension buffer containing 0.1% Tween 20, cells were pelleted and resuspended in 50 μL transposition mix (25 μL 2× TD buffer, 2.5 μL transposase (100 nM final), 16.5 μL PBS, 0.5 μL 1% digitonin, 0.5 μL 10% Tween 20, 5 μL H_2_O) and incubated for 30 min at 37 °C with shaking. The reaction was purified using the Zymo DNA Clean & Concentrator kit and PCR-amplified with NEBNext High-Fidelity 2× PCR Master Mix (NEB, M0541L) and primers as defined in Corces et al.^39^ Libraries were purified by two rounds of double-sided size selection with AMPure XP beads (Beckman Coulter, A63881), with the initial round of 0.5× sample volume of beads followed by a second round with 1.3× initial volume of beads. Library size distributions were confirmed by separation on a PAGE gel and staining with SYBRGold and pooled on the basis of quantifications from Qubit dsDNA High Sensitivity Kit. Pooled libraries were sequenced using the Novaseq 6000 platform (2 × 150 bp).

#### Luciferase assays

CNCCs were transfected with the appropriate plasmids immediately following passaging to passage 5 in 48-well plates. For *TWIST1-*tagged CNCCs, dTAG^V^-1 treatment to titrate TWIST1 dosage were started at the time of transfection, whereas for *SOX9-*tagged CNCCs, dTAGV-1 treatment was started 24h prior to passaging to p5 and transfection. Four independent transfections were performed for each dTAG^V^-1 concentration, with each well receiving 5ng of pGL3 plasmid, 0.25ng of control pRL firefly renilla plasmid, 44.25 μL carrier DNA (circularized pUC19 plasmid) and 0.3 μL Fugene 6 in 25 μL of optimum. The pGL3 plasmid contains the firefly luciferase gene driven by an SV40 promoter with either a control SV40 enhancer downstream, or a test enhancer sequence cloned upstream (Promega), the pRL plasmid acts as a transfection control with Renilla luciferase driven by an upstream CMV enhancer and CMV promoter (Promega). Test enhancers were cloned by either PCR of genomic DNA with primers containing NheI and XhoI restriction sites, or synthesized by Twist Biosciences with NheI and XhoI flanking restriction sites, and ligated into NheI and XhoI-digested pGL3 vector. 24 h after transfection, cells were washed in PBS, and lysed in 65 μL 1X passive lysis buffer (in PBS) for 15 min (Promega). 20 μL lysate was then transferred to an opaque flat-bottomed plate for reading with a luminometer (Veritas). An automated injector added 100 μL LARII reagent and the well was read using the following parameters: 2 s delay, 10 s integration. 100 μL Stop-and-Glow reagent was then injected into the well and read using the same parameters. Empty vector and the EC1.45 min1-2 enhancer (shown to be strongly active in CNCCs by Long et al.^40^) were included in each experiment as negative and positive controls, respectively.

### Quantification and statistical analysis

#### Statistical details and tests

Details of all statistical tests can be found in figures and figure legends. All boxplots represent the median (middle line), 25th and 75^th^ percentiles (hinges), and 1.5 times the interquartile range (whiskers).

#### ATAC-seq preprocessing

Reads were trimmed of Nextera adapter sequences and low-quality bases (-Q 10) using skewer^41^ v0.2.2 and then mapped to the hg38 analysis set (human) using Bowtie2^42^ v2.4.1 with the options --very-sensitive -X 2000. Reads were deduplicated with samtools^43^ v1.10 markdup and uniquely mapped reads (-q 20) mapped to the main chromosomes (excluding mitochondria and unplaced contigs) were retained using samtools view. Read ends were shifted inward 5 bp (+5 bp on + strand, -5bp on – strand) for each fragment, and then counts of reads in each sample overlapping the reproducible peak set of 151,457 REs from Naqvi et al.^11^ were generated using bedtools.^44^

#### Modeling of TF dose-response curves

TWIST1-dependent REs were defined by differential accessibility between undepleted and fully depleted TWIST1 concentrations was carried out using DESeq2^45^ v1.32.0, with CNCC differentiation batch as a covariate and raw counts as input. SOX9-dependent REs from Naqvi et al.^11^ were used. RE ATAC counts per million (CPM) values were first TMM-normalized using the edgeR package^46^ v3.34.0. For each TWIST1/SOX9-dependent RE, were corrected for differentiation batch effect by linear regression using the lm() function. Differentiation-corrected CPM values were scaled by dividing by the maximum absolute value across samples. Sample outliers, defined as *Z* score greater than 3, were removed from the analysis of that RE/gene. The data were then to the Hill equation using the drm() function in the drc R package^47^ v3.0-1. A two-parameter Hill equation (that is, with minimum and maximum fixed as the mean CPM at full or no depletion, respectively) was used unless a three-parameter Hill equation with fixed minimum but free maximum was a better fit (decrease in AIC > 2 relative to the two-parameter model); for these genes/REs, the three-parameter Hill was used. The Hill exponent as fitted was extracted, but for ED_50_ we used a modified calculation, calculating the TF dosage at which the normalized ATAC signal reached 50% of the signal at 100% TF dosage (rather than the theoretical maximum). This essentially caps the ED_50_ at 100% and avoids instability in high ED_50_ estimates especially when the three-parameter Hill was used.

#### Definition of training, testing, and validation sets

For predicting effect of full TF depletion, all 151,457 RE peaks were used and divided into training, test, and validation sets based on the chromosome-level “fold 0” training, testing, and validation split from the ChromBPNet^22^ package. For predicting ED_50_, the same fold split was used but only among the likely direct target REs of each TF. For SOX9 this was the “Rapid down” class of REs from Naqvi et al.^11^ (i.e., downregulated in accessibility after 3h full SOX9 depletion), and for TWIST1 this was defined as REs bound by TWIST1 ChIP-seq and downregulated at 24h.

#### Baseline prediction of RE responsiveness

For baseline approaches to predicting effect of full TF depletion or ED_50_, we encoded sequence information by quantifying known PWM matches in a 200 bp window around each RE ATAC peak summit. PWMs were obtained from the HOCOMOCO v11 core PWM set,^48^ and matched to sequences using MOODS^49^ v1.9.4.1 with -p 0.01 as a permissive cutoff. For each RE, the best PWM match (as determined by the highest MOODS match score) was stored and quantified with the MOODS match score. PWMs with no reported match were set to 0. GC and CpG content as well as unperturbed ATAC-seq signal (quantified as log_10_(baseMean) output from the DESeq model) were added as additional predictors. The matrix of predictors for the training and test sets were separately centered and scaled to have mean 0 and standard deviation 1. LASSO regression was performed using the cv.glmnet package in r with alpha 0.01 and nlambda 50. Random forest regression was performed with the randomForest package in r with ntree 100.

#### Deep learning model pretraining and fine-tuning

The pretrained deep learning model was obtained by running the full ChromBPNet v0.1.1 pipeline with default parameters on a consolidated BAM file of all unperturbed TF ATAC-seq samples. By default, ChromBPNet predicts base-resolution accessibility over 1000bp regions; due to the series of convolutional layers used in the model, making accurate predictions at the edges of these 1000bp regions requires considering the additional ∼500 bp flanking the edges, thus the receptive field is > 2000 bp. The 151,457 RE peak set from Naqvi et al.^11^ was used and a corresponding background peak set was created using chrombpnet prep nonpeaks. The “fold 0” training, testing, and validation split was used. Next, the model was fine-tuned with the effect size of full TF depletion or ED_50_ from the relevant training set. Learning rate was set to 1e−3 as in the original ChromBPNet training, with training for 10 epochs. The best-performing model (lowest loss on the validation set) was used. The same loss functions as the pretrained model were used, except the weight for the multinomial NLL loss (for the base-resolution profiles) was set to 0. Reverse-complemented sequences were used as data augmentation.

#### Model interpretation and motif matching

Contribution scores for both the pretrained and fine-tuned ChromBPNet models were extracted using chrombpnet contribs_bw with -pc counts, and TF-MoDISCO^24^ was run for motif discovery using the chrombpnet modisco_motifs command with -N 1000000. Top CWMs output from TF-MoDISCO were matched to their genomic locations by adapting a previously described procedure^6^^,^^7^ that considers both the Jaccardian similarity between a CWM and a test sequence as well as that sequence’s overall contribution score. As in Brennan et al.,^6^ because we were interested in CWM matches corresponding to low-affinity motifs, mapping thresholds were lowered to mapping the motif if the CWM Jaccard similarity percentile was equal to or greater than 10% and if the total absolute contribution percentile was equal to or greater than 0.5%. After mapping, motifs were filtered for redundant assignment of palindromic sequences and overlapping peaks; if multiple different CWMs matched the same sequence (as was frequent with partial and degenerate Coordinators), the CWM with the highest Jaccard similarity score (multiplied by CWM length to account for the fact that higher match scores are more likely with short motifs) was chosen.

#### Analyses of evolutionary constraint

Basepair-level PhastCons and phyloP scores from alignment of 30 primate genomes were obtained from https://hgdownload.soe.ucsc.edu/goldenPath/hg38/phastCons30way/and https://hgdownload.soe.ucsc.edu/goldenPath/hg38/phyloP30way/respectively, and averaged over buffering or sensitizing CWM occurrences as indicated. PhastCons and phyloP scores from alignment of 100 vertebrate genomes were obtained from https://hgdownload.soe.ucsc.edu/goldenPath/hg38/phastCons100way/and https://hgdownload.soe.ucsc.edu/goldenPath/hg38/phyloP100way/, respectively. The shuffled CWM occurrence set was generated with bedtools shuffle on a sufficiently large number of arbitrary regions within the set of TWIST1/SOX9-dependent REs, and then subtracting the true CWM occurrences. Selection estimates based on both within-species polymorphism and between-species divergence were obtained from the INSIGHT^26^ web tool (http://compgen.cshl.edu/INSIGHT/).

#### Analyses of nucleosome occupancy and positioning

NucleoATAC^29^ was run separately on aggregated.bam files from all replicates of a given TWIST1 dosage. Aggregate.bam files were downsampled to the least deeply sequenced dosage. ∗_nucmap_combined.bed.gz files were combined from each NucleoATAC run and distance to the closest CWM motif occurrences was calculated by bedtools closest. Only inferred nucleosomes with at least two supporting reads from each dosage were analyzed further.

#### Modeling of chromatin accessibility

The nucleosome-mediated cooperativity (NMC) model proposed by Mirny^28^ considers a DNA region that is either in a nucleosome-bound or open state. Assuming that there are n number of transcription factor binding sites in this region, the transcription factor can either bind in the nucleosome-bound or open state. However, there is a suppression of transcription factor binding in the nucleosome-bound state due to the energy cost of DNA unwrapping (i.e., TF-nucleosome competition). In the simplest form, there are three dimensionless parameters to describe the system: the equilibrium between nucleosome-bound and open state in the absence of transcription factors denoted as L, TF-nucleosome competition denoted as c, and effective protein concentration denoted as α. Using this model, the nucleosome occupancy (Y_N_) can be assayed as a function of protein concentration to assess chromatin accessibility. To study the effect of combining high-affinity and low-affinity transcription factor binding sites on accessibility, we assumed a region of DNA with a high- and a low-affinity sites. Accessibility is then calculated using the following equation:

$$Acc=1-Y_{N}=\frac{L\cdot \left(1+\alpha_{HA}\right)\cdot \left(1+\alpha_{LA}\right)}{\left(1+\alpha_{HA}\right)\cdot \left(1+\alpha_{LA}\right)+L\cdot \left(1+{c\alpha }_{HA}\right)\cdot \left(1+{c\alpha }_{LA}\right)}$$

Where α_LA_ and α_HA_ are the effective protein concentration for the strong and weak sites, defined as ${\alpha /K}_{D}^{HA}$ and ${\alpha /K}_{D}^{LA}$. The model parameters were set to L = 10^3^, c = 0 or 0.01 or 0.1, and α was titrated between 10^−5^ to 10^5^ to calculate the ED_50_ of the dosage response curves. To fit observed accessibility dosage response curves to the above model, python’s SciPy curve_fit library was used. First, the ED_50_ and Hill coefficient from the reporters containing only high-affinity sites was used to generate response curves that were fitted to the following equation:

$$Acc=1-Y_{N}=\frac{{\left(1+\alpha_{HA}\right)}^{n}}{{\left(1+\alpha_{HA}\right)}^{n}+L\cdot {\left(1+{c\alpha }_{HA}\right)}^{n}}$$

L was fixed at 10^3^. The mean squared error of the fit was calculated for n of 1–7 and it was determined that optimal fitting is achieved for *n* > 2, and thus we chose n^HA^ = 3. Next, the REs containing high- and low-affinity sites was fit to the following equation:

$$Acc=1-Y_{N}=\frac{{\left(1+\alpha_{LA}\right)}^{n^{LA}}\cdot {\left(1+\alpha_{HA}\right)}^{n^{HA}}}{{{\left(1+\alpha_{LA}\right)}^{n^{LA}}\cdot \left(1+\alpha_{HA}\right)}^{n^{HA}}+L\cdot {{\left(1+\alpha_{LA}\right)}^{n^{LA}}\cdot \left(1+{c\alpha }_{HA}\right)}^{n^{HA}}}$$

Where n^LA^ refers to the modeled number of low-affinity sites and is either matched to the number of sensitizing elements present in that RE, or is fixed at different values for all REs. Poor model fits (mean squared error >0.001 or negative fitted values of c) were removed from further analysis.

To study the effect of combining different transcription factor binding sites on chromatin accessibility, as is the case for SOX9 (Figure 3), we assumed the binding of at least two types of TFs:

$$Acc=\frac{{\left(1+\alpha_{TF1}\right)}^{n_{1}}\cdot {\left(1+\alpha_{TF2}\right)}^{n_{2}}}{{\left(1+\alpha_{TF1}\right)}^{n_{1}}\cdot {\left(1+\alpha_{TF2}\right)}^{n_{2}}+L\cdot {\left(1+c\alpha_{TF1}\right)}^{n_{1}}\cdot {\left(1+c\alpha_{TF2}\right)}^{n_{2}}}$$

Where $\alpha_{TF1}$ and $\alpha_{TF2}$ are the effective protein concentrations of the two transcription factors, and $n_{1}$ and $n_{2}$ are the number of binding motifs for each transcription factor. Assuming that there is only one binding motif for each transcription factor, the ED_50_ of the dosage response curve for one transcription factor can be analytically calculated assuming the other one stays constant:

$$\alpha_{TF1-ED50}=\frac{\left(1+\alpha_{TF2}\right)+L\left(1+{c\alpha }_{TF2}\right)}{\left(1+\alpha_{TF2}\right)+Lc\left(1+{c\alpha }_{TF2}\right)}$$

It can be proven that for c < 1, $\alpha_{TF1-ED50}$ is decreasing with increasing $\alpha_{TF2}$, showing the buffering effect of adding other transcription factor motifs in an RE. To fit observed SOX9 accessibility dosage response curves to the above model, the ED_50_ and Hill coefficient from the REs with a single SOX9 motif was used to generate response curves that were fitted to the following equation:

$$Acc=\frac{{\left(1+\alpha_{SOX9}\right)}^{n}}{{\left(1+\alpha_{SOX9}\right)}^{n}+L\cdot {\left(1+c\alpha_{SOX9}\right)}^{n}}$$

L was fixed at 10^3^. The mean squared error of the fit was calculated for n of 1–7 and it was determined that optimal fitting is achieved for *n* = 2. Next, the REs containing SOX9 and either AP2⍺ or coordinator motif were fitted to the following equation:

$$Acc=\frac{{\left(1+\alpha_{SOX9}\right)}^{2}\cdot \left(1+\alpha_{TF2}\right)}{{\left(1+\alpha_{SOX9}\right)}^{2}\cdot \left(1+\alpha_{TF2}\right)+L\cdot {\left(1+c\alpha_{SOX9}\right)}^{2}\cdot \left(1+{c\alpha }_{TF2}\right)}$$

Poor model fits (mean squared error >0.01 or negative fitted values of c) were removed from further analysis.

## Supplemental information

Document S1. Figures S1–S11

Table S1. Statistics and metadata for TWIST1-dependent REs, related to Figure 2

Table S2. Statistics and metadata for SOX9-dependent REs, related to Figure 2

Table S3. Contribution scores and additional statistics for CWM instances contributing to TWIST1 dosage response, related to Figure 3

Table S4. Contribution scores and additional statistics for CWM instances contributing to SOX9 dosage response, related to Figure 3

Table S5. Comparison of dosage response between endogenous accessibility and enhancer reporter activity for wild-type sequences, related to Figure 5

Table S6. Sequences and TWIST1/SOX9 dosage responses of wild-type and mutant enhancers, related to Figure 5

Document S2. Article plus supplemental information
