# Supplementary material for: Transfer learning reveals sequence determinants of the quantitative response to transcription factor dosage
Source: Cell Genom. 2025 Feb 27;5(3):100780. doi: 10.1016/j.xgen.2025.100780 (PMC11960506; doi:10.1016/j.xgen.2025.100780)
Supplement: Document S1. Figures S1–S11 [file mmc1.pdf]

Cell Genomics, Volume *5*

Supplemental information

Transfer learning reveals sequence determinants of the quantitative response to transcription factor dosage

Sahin Naqvi, Seungsoo Kim, Saman Tabatabaee, Anusri Pampari, Anshul Kundaje, Jonathan K. Pritchard, and Joanna Wysocka

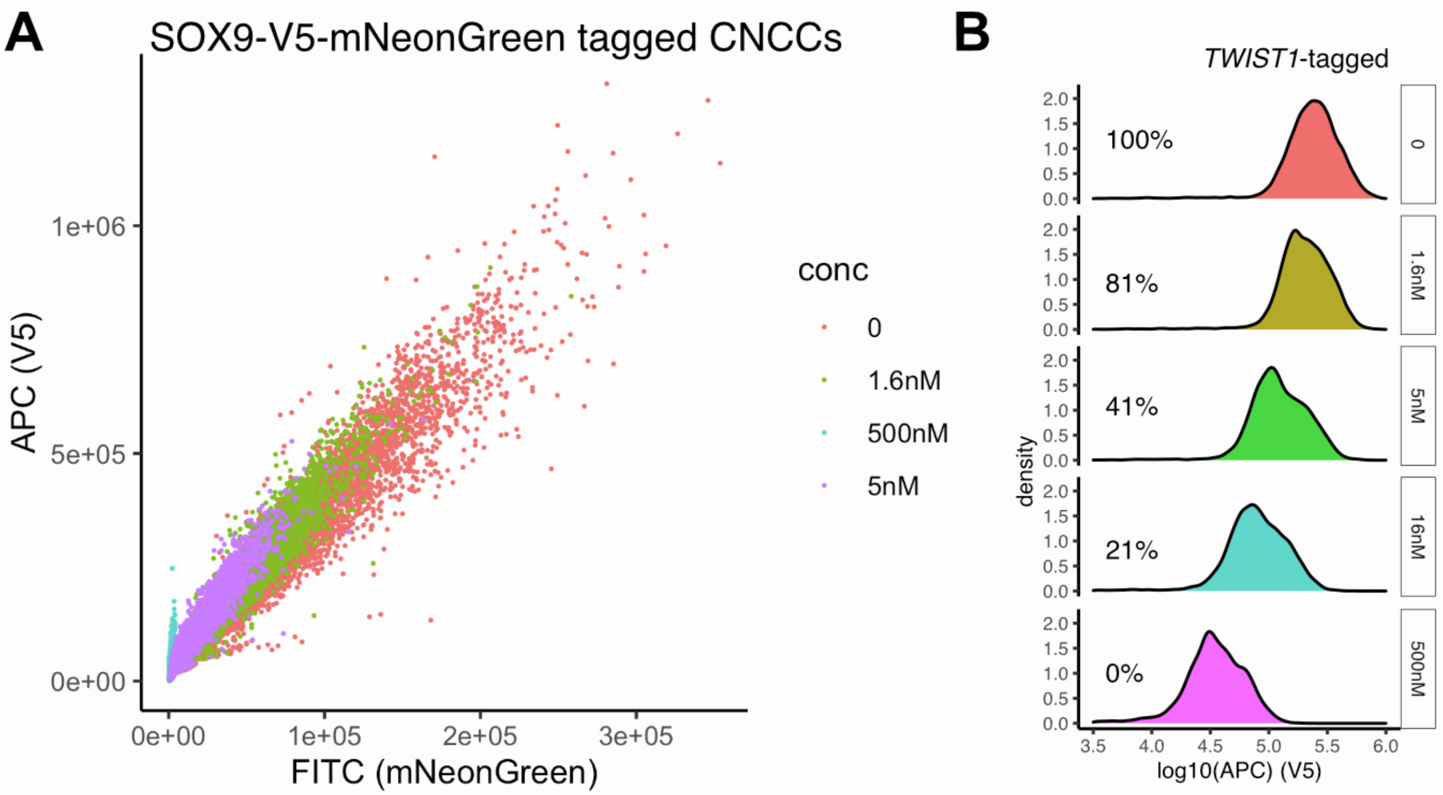

Figure S1. Precise modulation of TWIST1 dosage, related to Figure 1. (A) Comparison of V5 (y axis) and mNeonGreen (x-axis) signal in single SOX9-tagged cells treated with different dTAGV-1 concentrations. (B) Second independent replicate of TWIST1 dosage modulation, as in Figure 1B (at least 5,000 cells per histogram).

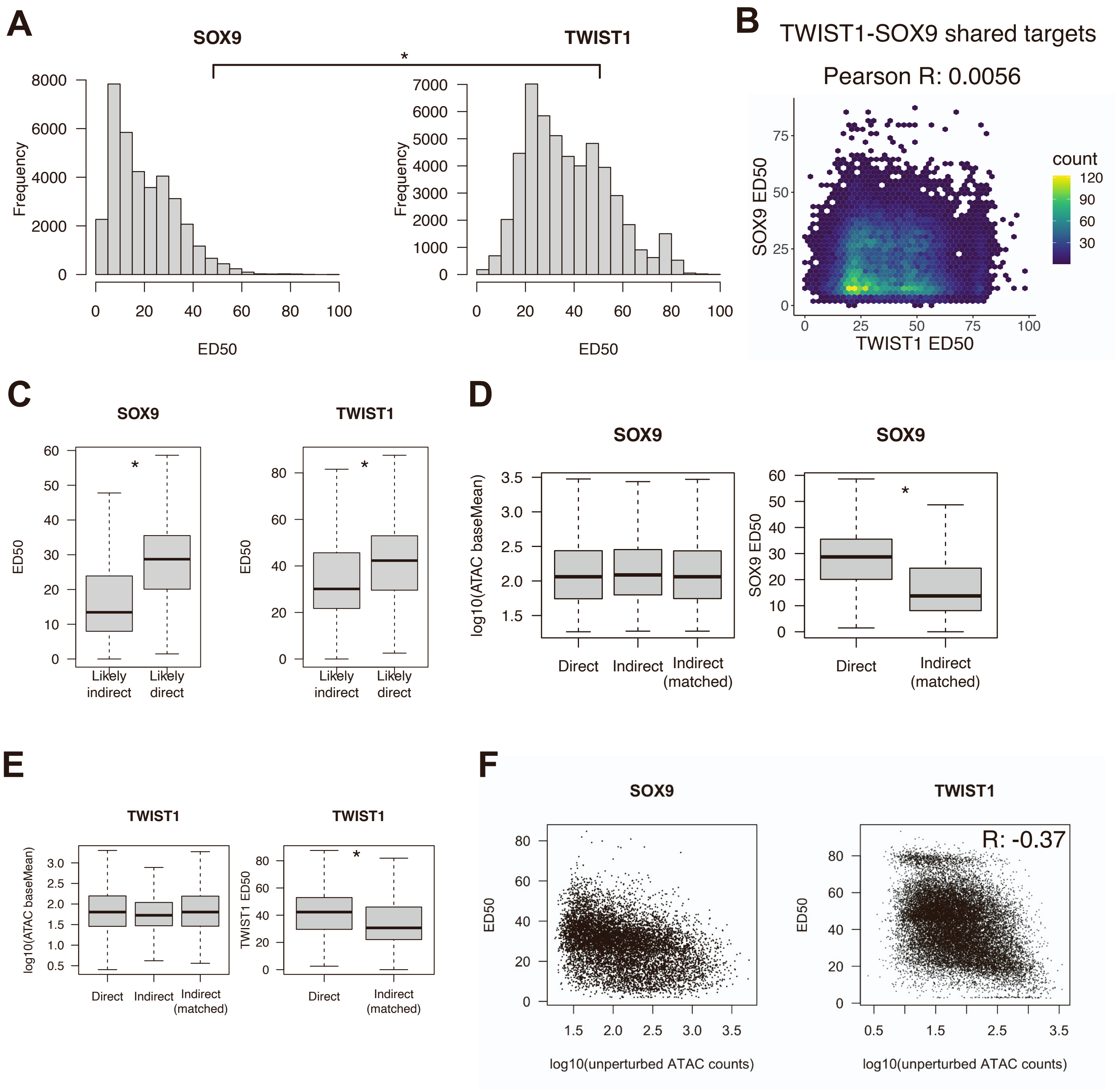

Figure S2. RE sensitivity to SOX9 and TWIST1 dosage, related to Figure 2. (A) Distribution of ED50 values among all SOX9 or TWIST1-dependent REs. (B) ED50 with respect to SOX9 dosage (y-axis) and TWIST1 dosage (x-axis) for all REs that are both SOX9- and TWIST1-dependent. (C) ED50 of likely direct or indirect SOX9 or TWIST1 targets. For SOX9, likely direct targets (*n*=9,279) were defined as the 3h downregulated class as in Naqvi et al 2023 (*n*= 26,434 indirect), and for TWIST1, direct targets (*n*=29,686) were defined as downregulated and containing a TWIST1 ChIP-seq peak. (D,E) Same sets of REs as in (C) but matching unperturbed ATAC-seq counts between direct and indirect target sites. (F) Unperturbed accessibility (x-axis) versus ED50 (y-axis) for all likely direct SOX9 or TWIST1 targets. * $p < 2.2e-16$, two-sided Wilcoxon rank-sum test.

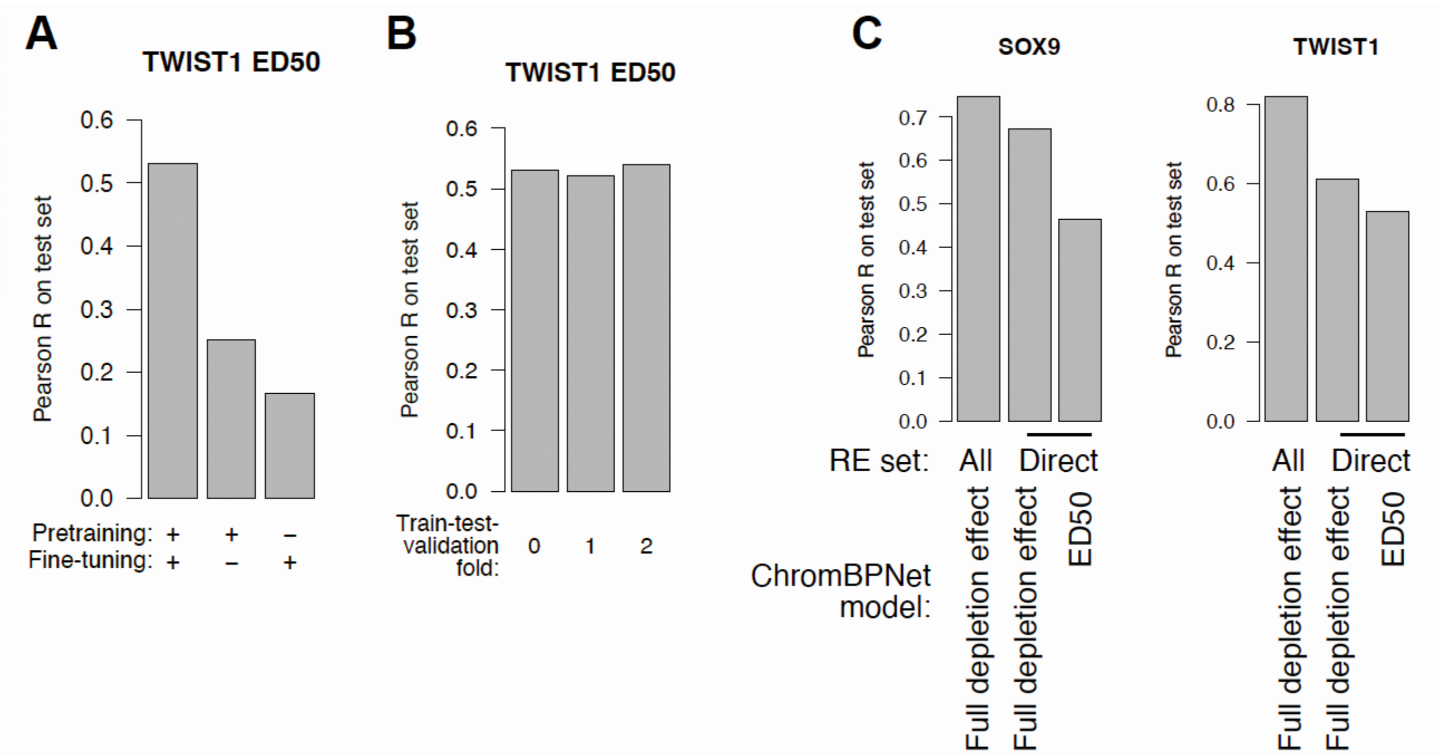

Figure S3. Prediction of effect of full TF depletion and ED50 from DNA sequence, related to Figure 2. (A) Prediction accuracy of TWIST ED50 prediction with and without pretraining or fine-tuning of ChromBPNet model. (B) Performance of pretrained and fine-tuned ChromBPNet model for predicting TWIST1 ED50 across three independent train-test-validation splits. (C) Decreased performance of ChromBPNet model for prediction effect of full SOX9 (left) or TWIST1 (right) depletion when predictions are subsetted to only direct RE targets (middle bar in each plot).

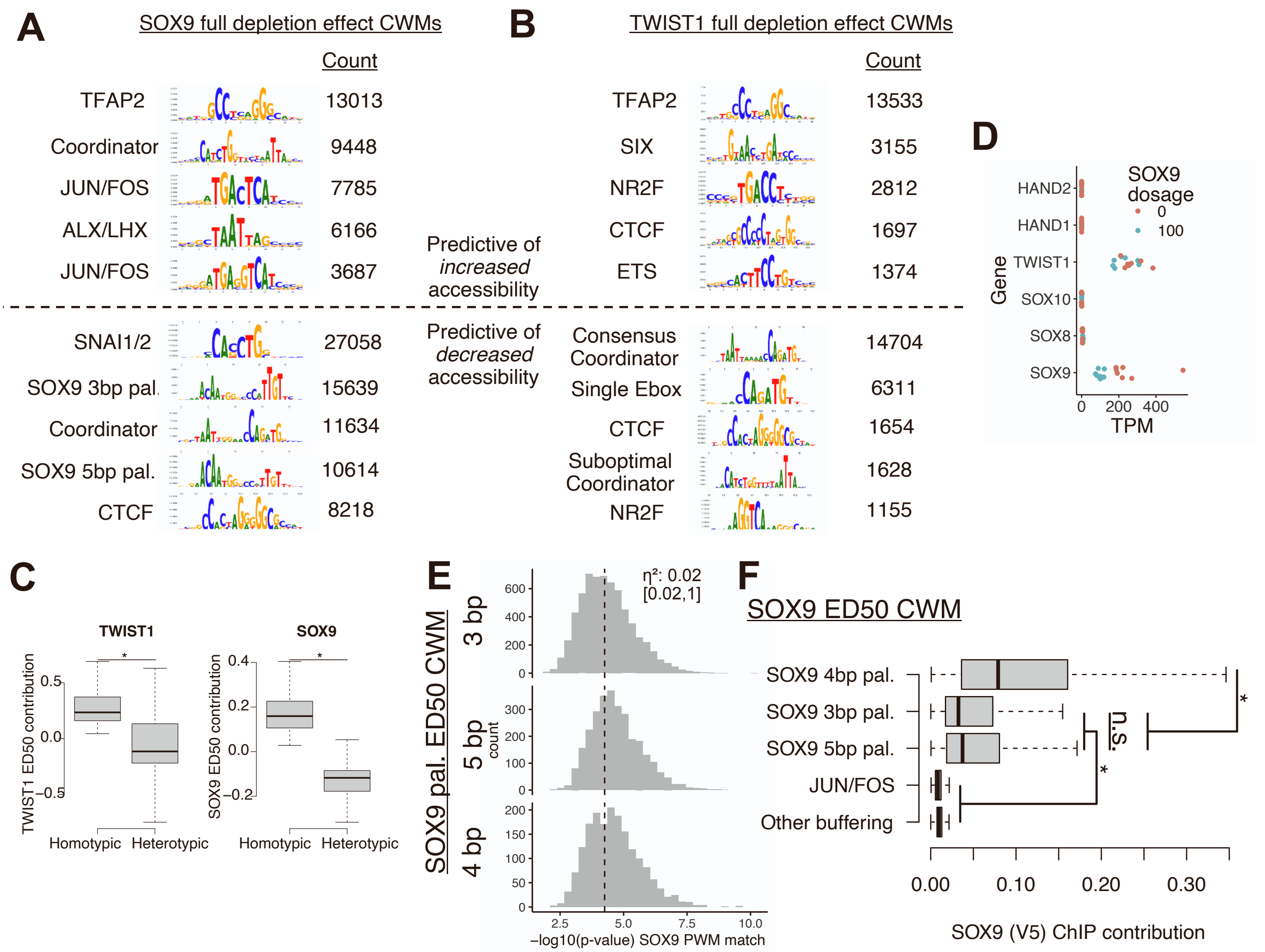

Figure S4. Sequence features predictive of the effect of full TF depletion on RE accessibility, related to Figure 3. (A,B) Top contribution weight matrices (CWMs) predictive of effect of full depletion of (A) SOX9 or (B) TWIST1 on RE accessibility. Number of individual occurrences of each CWM is indicated under the "count" column. (C) Comparison of contribution to TWIST1 (left) or SOX9 (right) ED50 for all CWMs with homotypic or heterotypic binding motifs. * $p < 2.2e-16$, two-sided Wilcoxon rank-sum test. *n* for groups: TWIST1 Homotypic 20,261; TWIST1 Heterotypic 42,003; SOX9 Homotypic 11,088; SOX9 Heterotypic 11,581. (D) Expression (transcripts per million) of indicated genes (y-axis) at full SOX9 dosage and complete loss (colors). Each point is one biological replicate. (E) For all individual instances of the indicated CWMs predictive of SOX9 ED50 (rows), the strength of that sequence match to SOX9 palindrome position weight matrix (PWM) is shown (x-axis). η^2 and 95% confidence interval in brackets from ANOVA of -log10(PWM match) as a function of SOX9 CWM palindrome type. (F) For the indicated CWMs, the distribution of contribution to SOX9 binding, estimated from BPNet on SOX9-V5 ChIP-seq, is shown. *n* of groups from top to bottom: 6,404, 2,887, 1,797, 915, 9,953. * $p < 2.2e-16$; n.s. $p < 0.05$, two-sided Wilcoxon rank sum test between each pair of the two groups indicated.

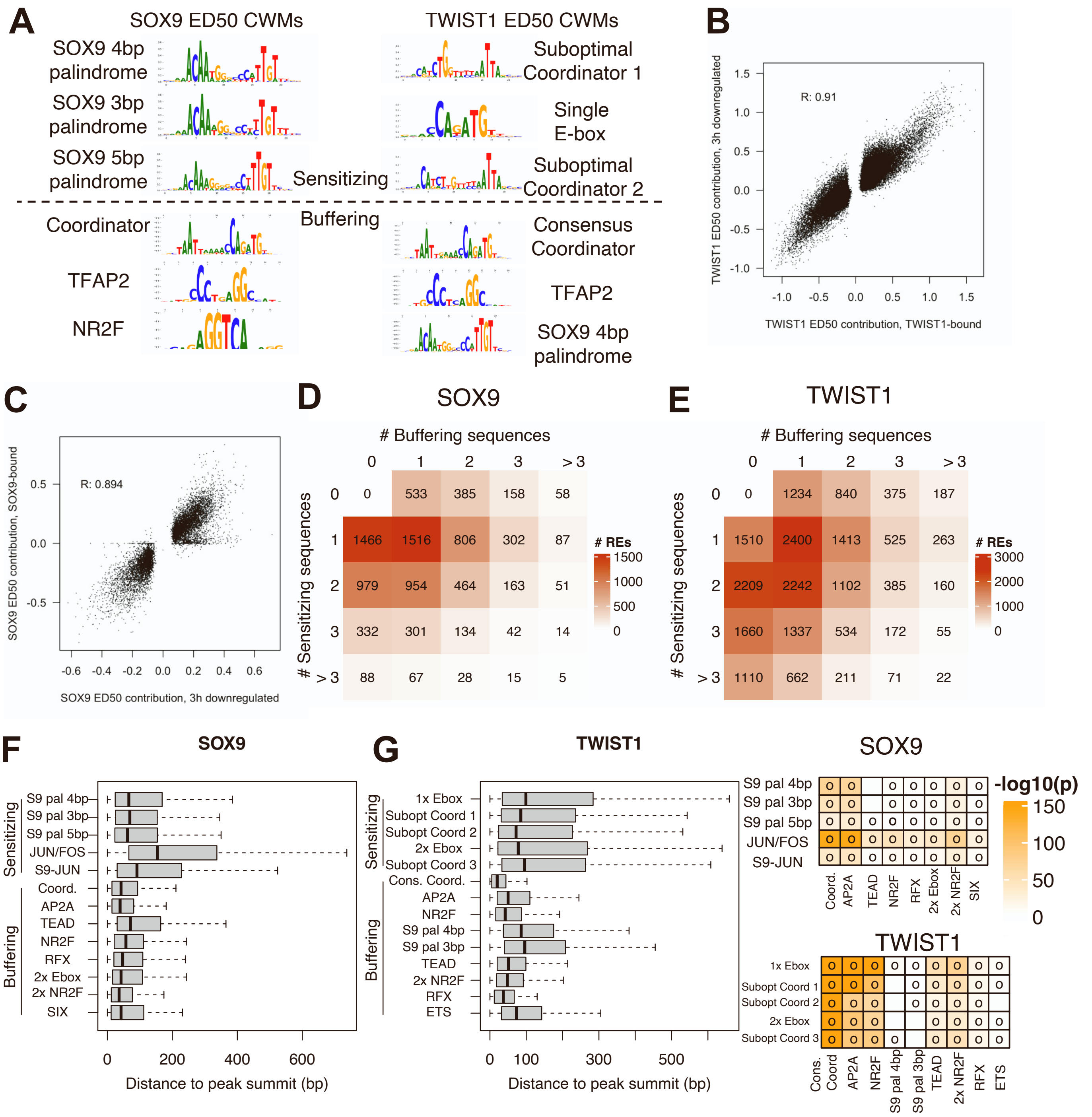

Figure S5. Robustness of results and additional features of buffering and sensitizing sequences, related to Figure 3. (A) Top contribution weight matrices (CWMs) predictive of SOX9 (left) or TWIST1 (right) ED50 using alternative definitions of direct targets for each TF (bound by ChIP-seq for SOX9, downregulated by 3h for TWIST1). (B) For all CWM occurrences, comparison of TWIST1 ED50 contribution scores when defining direct targets by 3h downregulated (y-axis) or TWIST1 binding by ChIP-seq (x-axis). (C) Same as (B) but for SOX9. (D,E) The number of (D) SOX9 or (E) TWIST1 target REs with the indicated number of buffering (x-axis) or sensitizing (y-axis) CWM occurrences. (F,G), Distance to ATAC peak summit of individual types of sensitizing or buffering CWM occurrences for SOX9 (F) or TWIST1 (G). Right, -log10(p-values) of two-sided Wilcoxon rank-sum test comparing distance to peak summit between indicated groups of sensitizing (y-axis) or buffering (x-axis) CWM occurrences for SOX9 (as in F) or TWIST1 (as in G). o, Benamini-Hochberg adjusted p-value < 0.05.

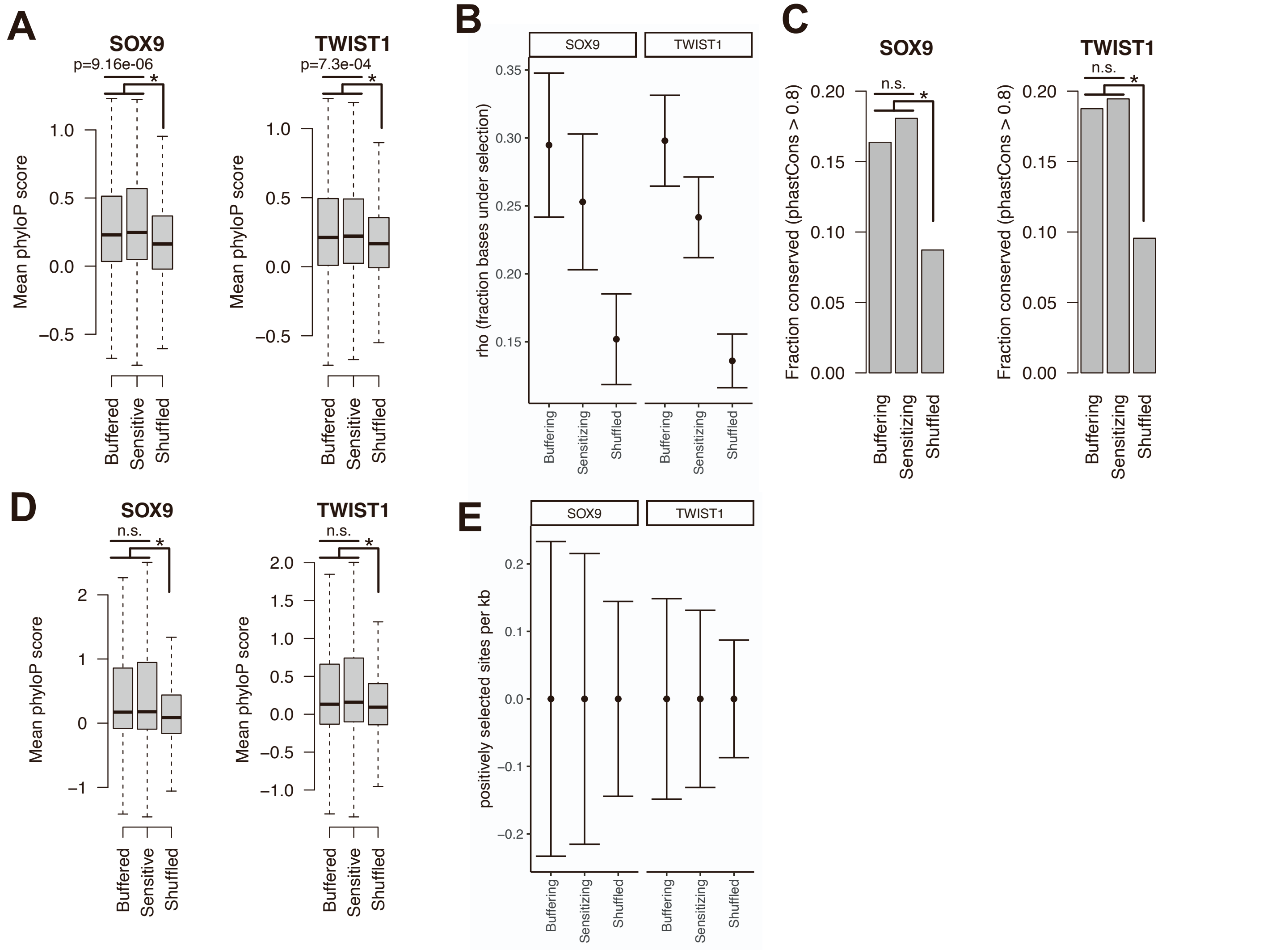

Figure S6. Signatures of selection at sensitizing and buffering sequences, related to Figure 4. (A) Mean phyloP score of buffering, sensitizing, or shuffled motif occurrences for SOX9 or TWIST1 ED50 estimate from primate genome alignments. Positive phyloP scores mean more likely to be conserved, negative means more likely to be under positive selection. (B) Fraction of sites under weak negative selection for the same classes of motifs as in (A), estimated by INSIGHT. Error bars indicate estimate +/- standard error. (C) Fraction of buffering, sensitizing, or location-shuffled occurrences showing evidence of evolutionary conservation estimated from vertebrate genome alignments for SOX9 (left) or TWIST1 (right). (D) phyloP scores as in (A) but estimate from vertebrate genome alignments (E) Frequency of sites under positive selection, estimated by INSIGHT. Error bars indicate estimate +/- standard error. *n* for groups: SOX9 buffering 9,953; SOX9 sensitizing 12,716; SOX9 shuffled 26,596; TWIST1 buffering 23,647; TWIST1 sensitizing 38,614; TWIST1 shuffled 71,012. * $p < 2.2e-16$, n.s. $p > 0.05$, Wilcoxon rank-sum test. Other p-values are shown.

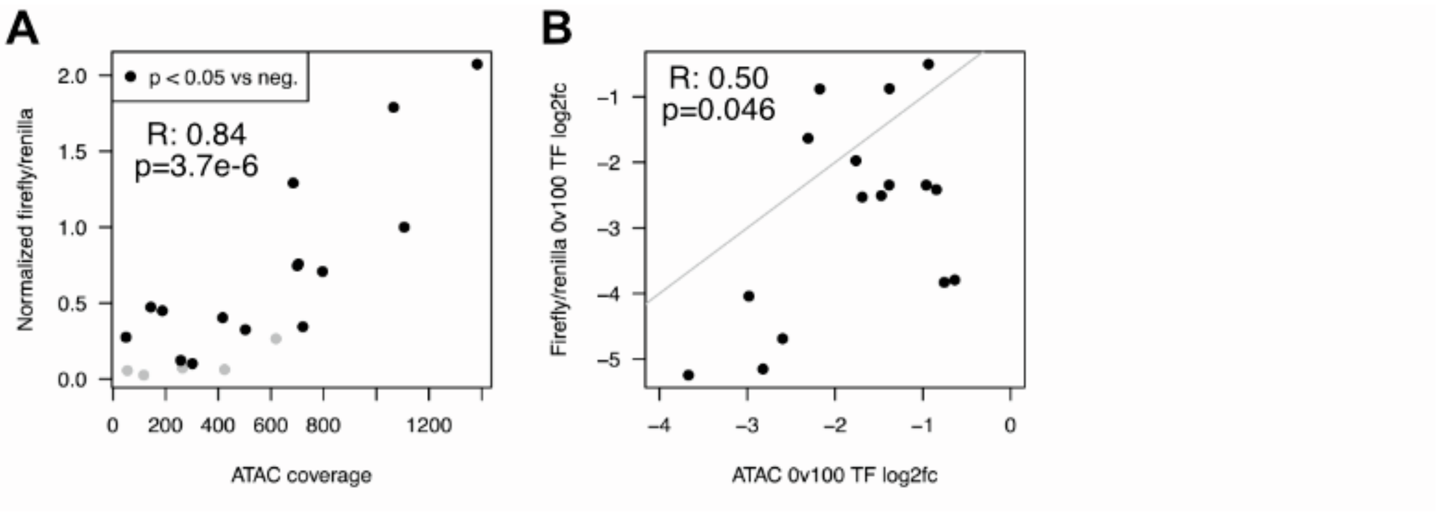

Figure S7. Dosage responses of wildtype REs in enhancer reporter assays, related to Figure 5. (A) Comparison of endogenous unperturbed accessibility (x-axis) and enhancer reporter activity (y-axis, normalized to positive control in each experiment) across 19 TWIST1- and SOX9-dependent REs (points). (B) For the REs in (A) with significantly higher activity than the negative control, the effect of full TF depletion on endogenous accessibility (x-axis) is compared to the effect of full TF depletion on enhancer reporter activity (y-axis). (C) Example of *MSR1* enhancer, where converting two high-affinity, buffering Coordinator motifs into a double E-box and low-affinity (LA) Coordinator motif has a sensitizing effect. (D) Comparison of changes relative to wildtype in reporter activity at 100% TF dosage (x-axis) and ED50 (y-axis) for tested mutant enhancers. (E) Example of *IMPG1* enhancer, where ectopically inserting five single E-box motifs at the indicated positions has a sensitizing effect

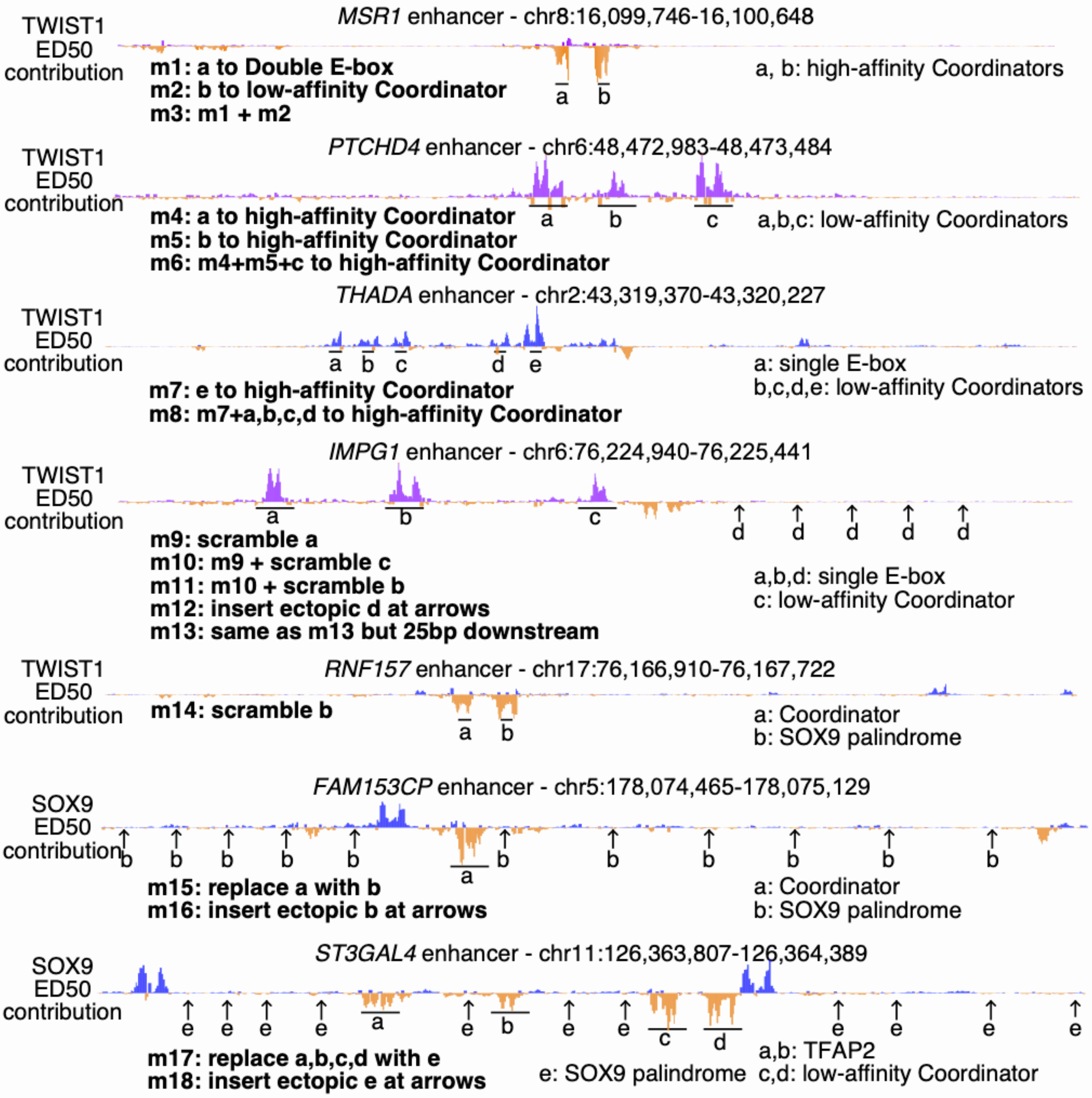

Figure S8. Schematic of mutant RE sequences, related to Figure 5. For each indicated RE, the TWIST1 or SOX9 ED50 contribution is plotted. Specific motif instances manipulated within each RE are labeled with letters. The changes using these endogenous or ectopic motif instances are indicated in bold for each mutant sequence designated with "m_."

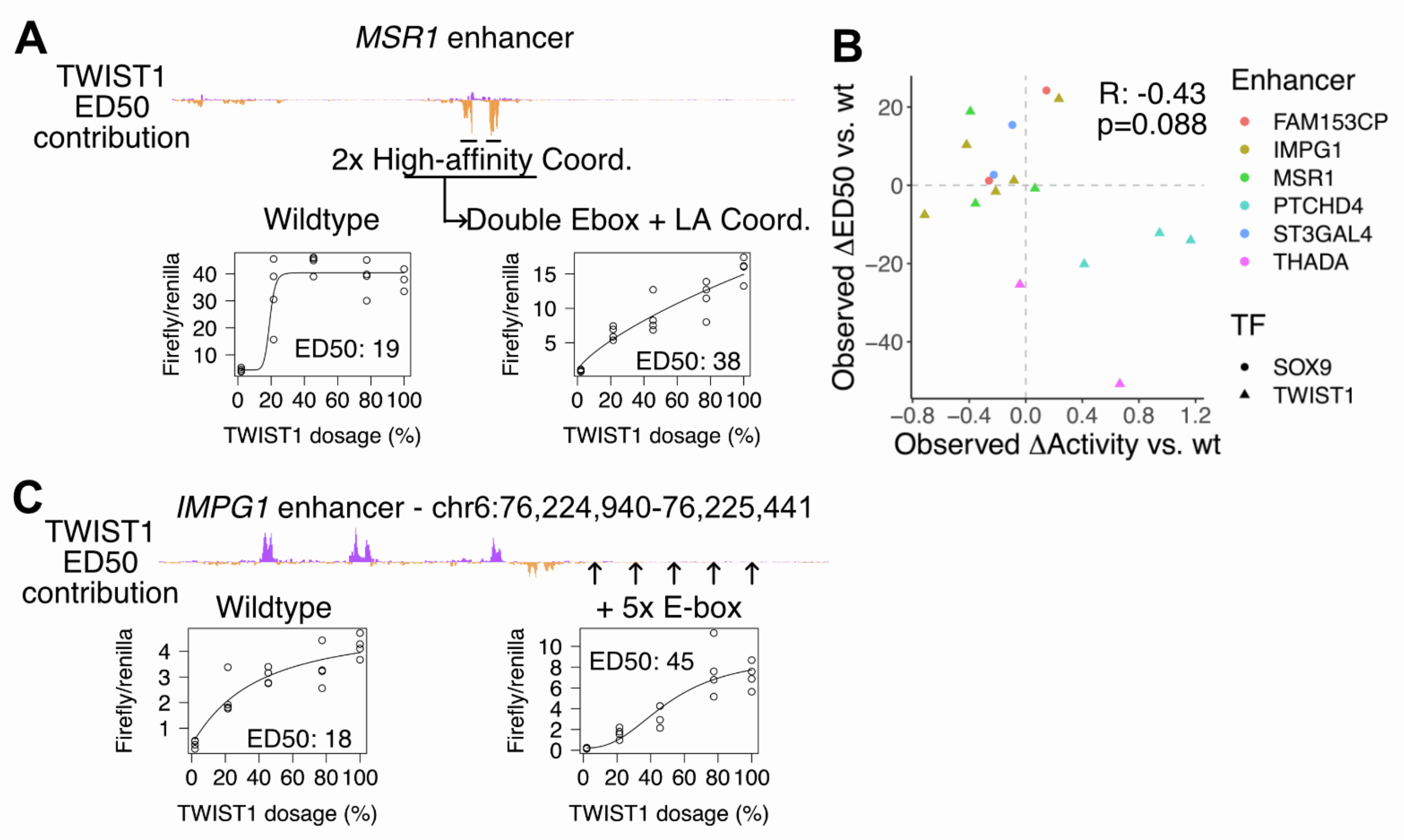

Figure S9. Dosage responses of mutant REs in enhancer reporter assays, related to Figure 5. (A) Example of *MSR1* enhancer, where converting two high-affinity, buffering Coordinator motifs into a double E-box and low-affinity (LA) Coordinator motif has a sensitizing effect. (B) Comparison of changes relative to wildtype in reporter activity at 100% TF dosage (x-axis) and ED50 (y-axis) for tested mutant enhancers. (C) Example of *IMPG1* enhancer, where ectopically inserting five single E-box motifs at the indicated positions has a sensitizing effect

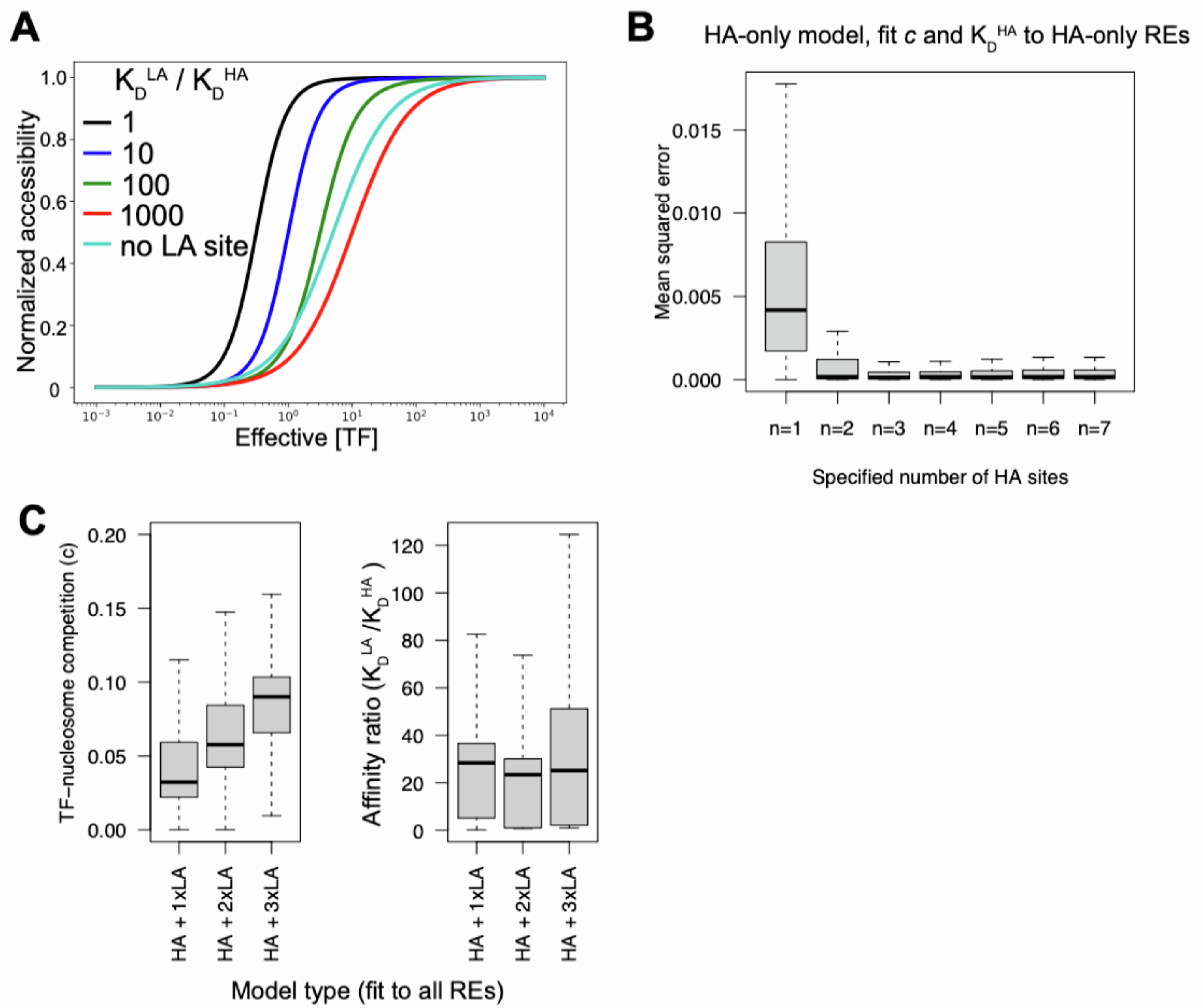

Figure S10. Theoretical and fitted instances of TF-nucleosome competition model, related to Figure 6. (A) Effect of low-affinity site (colors) or no site (grey) on theoretical dosage curves under model weak TF-nucleosome competition (*c* = 0.001). (B) Mean squared error of high-affinity (HA)-only model with specified effective number of HA sites (x-axis), fit to 1,291 REs. (C) Values of *c* or high/low-affinity site K_D obtained by fitting model to observed dosage response curves for REs a mix of high-and low-affinity Coordinator sites (HA + LA). All REs (*n* = 1,291) were fit with the indicated model rather than models matched to the number of LA REs in each.

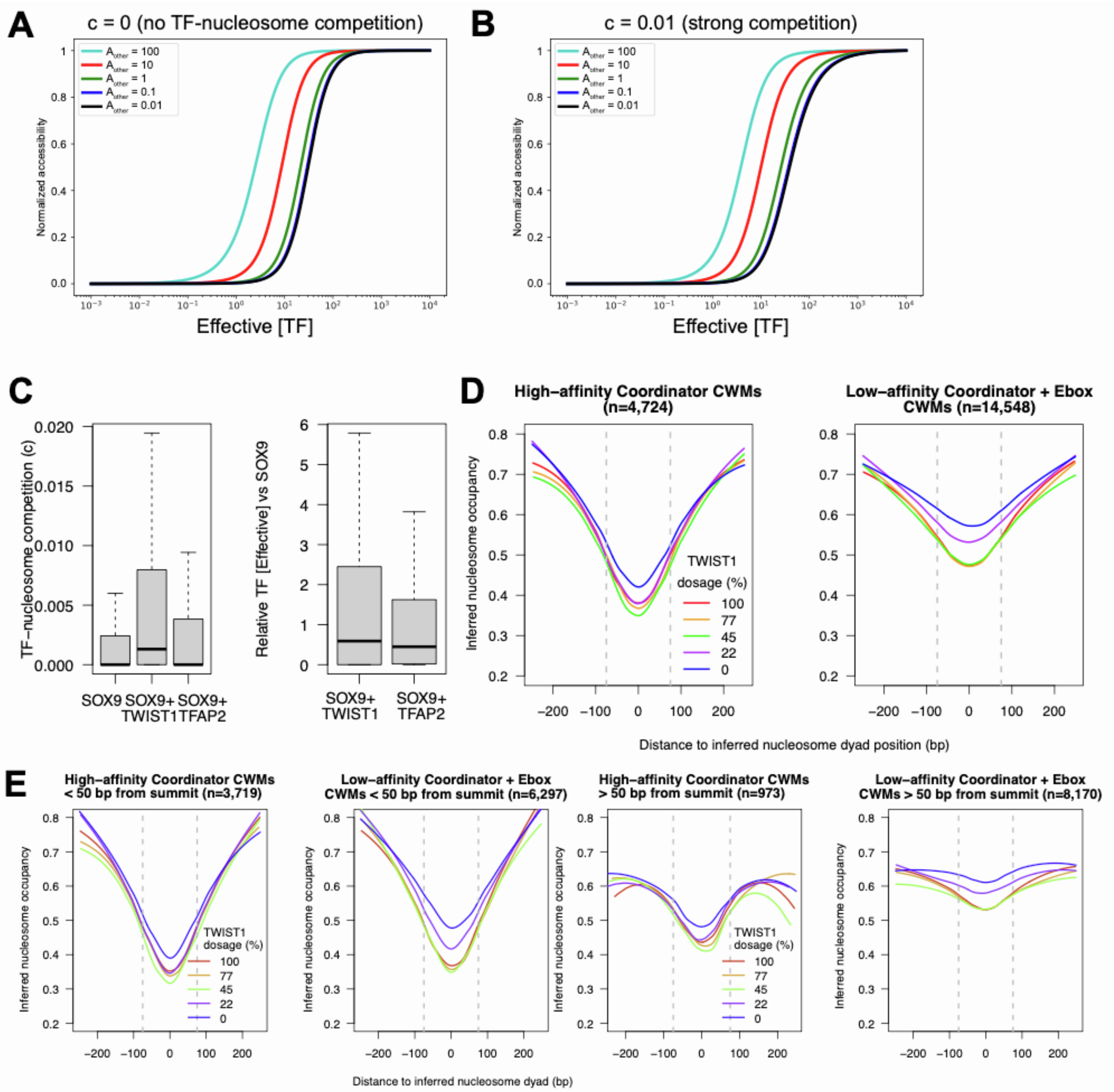

Figure S11. Modeling of heterotypic TF binding and analysis of nucleosome occupancy and positioning, related to Figure 6. (A,B) Effect of other, unperturbed TF effective concentration (colors) on theoretical dosage curves under no TF-nucleosome competition (left) or strong competition (right). (C) (left) Values of *c* obtained by fitting heterotypic TF binding model to REs with only SOX9 motifs (*n*=1,372), SOX9 and Coordinator motifs (*n*=686), or SOX9 and TFAP2 motis (*n*=386). (right) Relative effective concentration of the other TF from the same indicated models. (D) Loess fit of inferred nucleosome occupancy and dyad position relative to all high- (left) or low- (right) affinity motifs at different TWIST1 dosages (colors). (E) Same as (D), but motifs are split by location within REs (left two plots, within 50 bp of RE summit; right two plots, greater than 50 bp from RE summit)
